# Heterogeneity in allospecific T cell function in transplant-tolerant hosts determines susceptibility to rejection following infection

**DOI:** 10.1172/JCI168465

**Published:** 2023-11-01

**Authors:** Christine M. McIntosh, Jennifer B. Allocco, Peter Wang, Michelle L. McKeague, Alexandra Cassano, Ying Wang, Stephen Z. Xie, Grace Hynes, Ricardo Mora-Cartín, Domenic Abbondanza, Luqiu Chen, Husain Sattar, Dengping Yin, Zheng J. Zhang, Anita S. Chong, Maria-Luisa Alegre

**Affiliations:** 1 Department of Medicine, Section of Rheumatology,; 2 Department of Surgery, Section of Transplantation, and; 3 Department of Pathology, University of Chicago, Chicago, Illinois, USA.; 4 Comprehensive Transplant Center and; 5 Department of Surgery, Northwestern University Feinberg School of Medicine, Chicago, Illinois, USA.

**Keywords:** Transplantation, Adaptive immunity

## Abstract

Even when successfully induced, immunological tolerance to solid organs remains vulnerable to inflammatory insults, which can trigger rejection. In a mouse model of cardiac allograft tolerance in which infection with *Listeria monocytogenes* (Lm) precipitates rejection of previously accepted grafts, we showed that recipient CD4^+^ TCR75 cells reactive to a donor MHC class I–derived peptide become hypofunctional if the allograft is accepted for more than 3 weeks. Paradoxically, infection-induced transplant rejection was not associated with transcriptional or functional reinvigoration of TCR75 cells. We hypothesized that there is heterogeneity in the level of dysfunction of different allospecific T cells, depending on duration of their cognate antigen expression. Unlike CD4^+^ TCR75 cells, CD4^+^ TEa cells specific for a peptide derived from donor MHC class II, an alloantigen whose expression declines after transplantation but remains inducible in settings of inflammation, retained function in tolerant mice and expanded during Lm-induced rejection. Repeated injections of alloantigens drove hypofunction in TEa cells and rendered grafts resistant to Lm-dependent rejection. Our results uncover a functional heterogeneity in allospecific T cells of distinct specificities after tolerance induction and reveal a strategy to defunctionalize a greater repertoire of allospecific T cells, thereby mitigating a critical vulnerability of tolerance.

## Graphical abstract

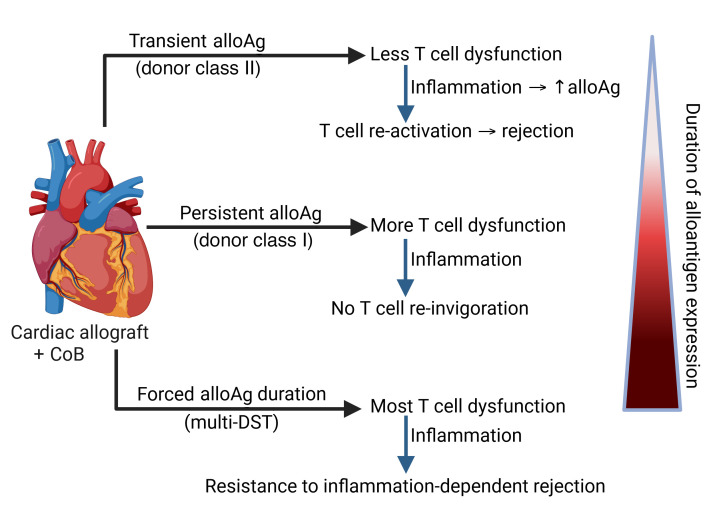

## Introduction

Achieving stable donor-specific transplantation tolerance holds the potential for vastly improving the quality of life for transplant recipients by eliminating the lifelong exposure to drug toxicity, higher risk of malignancy and infection, and development of chronic rejection associated with conventional immunosuppression. In mice, treatment with anti-CD154 (αCD154) and donor splenocyte transfusion (DST) produces a robust state of graft-specific tolerance to fully MHC-mismatched cardiac allografts (1) that, once established, resists many late inflammatory challenges such as TLR agonists or infections, including *lymphocytic choriomeningitis virus* (LCMV) (2) and *Staphylococcus aureus* (3). That these inflammatory challenges at the time of transplantation prevent the induction of tolerance (4) but cannot break established tolerance supports that αCD154/DST-induced tolerance is robust. However, we have shown that late infection of tolerant mice with the intracellular bacterium *Listeria monocytogenes* (Lm) precipitates T cell–dependent cardiac allograft rejection (5, 6). Lm-induced rejection was dependent on IL-6 and type I IFN, and the combination of IL-6 and IFN-β was sufficient to break established tolerance in the absence of infection, ruling out crossreactivity to graft antigens by Lm-reactive T cells and suggesting that tolerance may be vulnerable to inflammatory settings eliciting these cytokines (5). There is also circumstantial evidence that infection poses a threat to clinical transplantation tolerance. A subset of patients who spontaneously developed operational tolerance following cessation of conventional immunosuppression rejected their grafts after years of stability, often after an infection (7). Furthermore, reports of patients made tolerant to renal allografts with concurrent hematopoietic stem cell or bone marrow transplantation who later rejected their allografts following an infection suggest that even the most effective strategies currently available for inducing clinical tolerance leave grafts vulnerable to inflammatory challenges (8, 9).

Understanding the mechanisms of rejection after successful establishment of tolerance is important for developing strategies for improving the stability of tolerance. While many studies have focused on the mechanisms of acute allograft rejection in untreated mice, little is known about rejection in previously tolerant mice. In this study, we aim to understand the mechanisms by which Lm infection was able to precipitate T cell–dependent allograft rejection in tolerant mice (5). αCD154/DST-mediated tolerance is associated with induction of allospecific Tregs (10) and abortive proliferation of alloreactive conventional T cells (Tconvs), resulting in a high Treg/Tconv ratio (11). Using adoptively transferred allospecific TCR-transgenic T cells as a tracer of the endogenous alloresponse, our group previously found that TCR75 cells, TCR-transgenic CD4^+^ Tconvs specific for a donor MHC class I–derived (MHCI-derived) peptide indirectly presented by host MHCII, become antigen experienced and persist in tolerant graft recipients. However, in contrast to TCR75 cells in rejecting mice, which develop a phenotype and functional capacity consistent with memory T cells, TCR75 cells in tolerant mice take on a phenotype and hypofunctional profile combining features of exhausted and anergic T cells (12). Importantly, TCR75 cells did not appear to regain function after Lm infection when assessed a month after infection (12) despite Lm precipitating rejection (13) or eroding tolerance (6) in all previously tolerant hosts. Thus, it is unlikely that these profoundly hypofunctional allospecific Tconvs mediate rejection after infection of tolerant hosts. We therefore hypothesized that there are allospecific Tconv clones of other specificities that retain functionality and are thus more poised to reject the graft when a stably tolerant host becomes infected.

Parameters of T cell hypofunction have been identified in tolerant recipients with monoclonal tracer TCR75 cells as well as with endogenous allospecific T cells detected with allogeneic peptide:MHC (pMHC) multimers (12). However, the T cells tracked thus far are unlikely to be representative of the entire allospecific T cell population, which is estimated to contain over 1% of mature T cells (14). Variables including avidity for alloantigen and specificity for a particular alloantigen may affect the fate of allospecific T cells during tolerance. Duration of antigen presentation has recently become appreciated as a key variable modulating allospecific T cell responses during rejection (15, 16). Having determined that chronic alloantigen exposure is necessary for the development of hypofunction in allospecific TCR75 cells during αCD154/DST-induced tolerance (12), we investigated whether allospecific T cells specific for a less persistent alloantigen retain more functionality than TCR75 cells.

Previous findings from our group indicated that the graft must persist for approximately 3 weeks for tracer TCR75 cells to acquire hypofunction, as TCR75 cells were impaired functionally if grafts were surgically removed 3 weeks, but not 1 week, after transplantation in hosts treated with αCD154/DST (12). However, some alloantigens are not expressed persistently in the graft. Unlike donor MHCI (the source of cognate peptide for TCR75 cells), which is constitutively expressed by all nucleated cells and thus has the potential to drive persistent stimulation of direct and indirect alloreactive T cells, direct alloresponses to donor MHCII and indirect alloresponses to peptides derived from donor MHCII and presented by host MHC appear to be transient (15). Expression of donor MHCII antigens is thought to be short lived in the graft because donor antigen-presenting cells (APCs) accompanying the graft die shortly after transplantation (15). However, endothelial cells can upregulate expression of MHCII in response to IFN-γ, providing a source of donor MHCII antigen late after transplantation in settings of inflammation, for example, during an infection. Furthermore, any polymorphic inflammation-induced molecules may similarly act as transient sources of alloantigen, with reexpression later during inflammation. Thus, there is a potentially substantial population of alloreactive T cells specific for transiently expressed alloantigens whose fate during tolerance remains to be explored.

Here, we investigated functionality following the induction of tolerance of TEa cells transgenic for a TCR recognizing a donor MHCII–derived peptide presented indirectly by recipient MHCII. We found that these cells retained greater functionality than donor MHCI–derived peptide-reactive TCR75 cells. Notably, TEa cells could be made profoundly hypofunctional with repeated antigen stimulation over approximately 4 weeks. Furthermore, this treatment rendered grafts resistant to Lm-triggered rejection. Prolonging exposure to alloantigens through repeated alloantigen administration during tolerance induction represents a therapeutic approach for improving the robustness of tolerance by achieving hypofunction in a broader array of allospecific T cells. Our study reveals that heterogeneity in functional loss of distinct allospecific T cells is an important vulnerability to durable transplantation tolerance and that prolonged alloantigen exposure is a possible solution for ensuring greater graft stability in the face of inflammatory challenges.

## Results

### Donor MHCI peptide–reactive monoclonal T cells are not refunctionalized after Lm infection.

**Figure 1:**
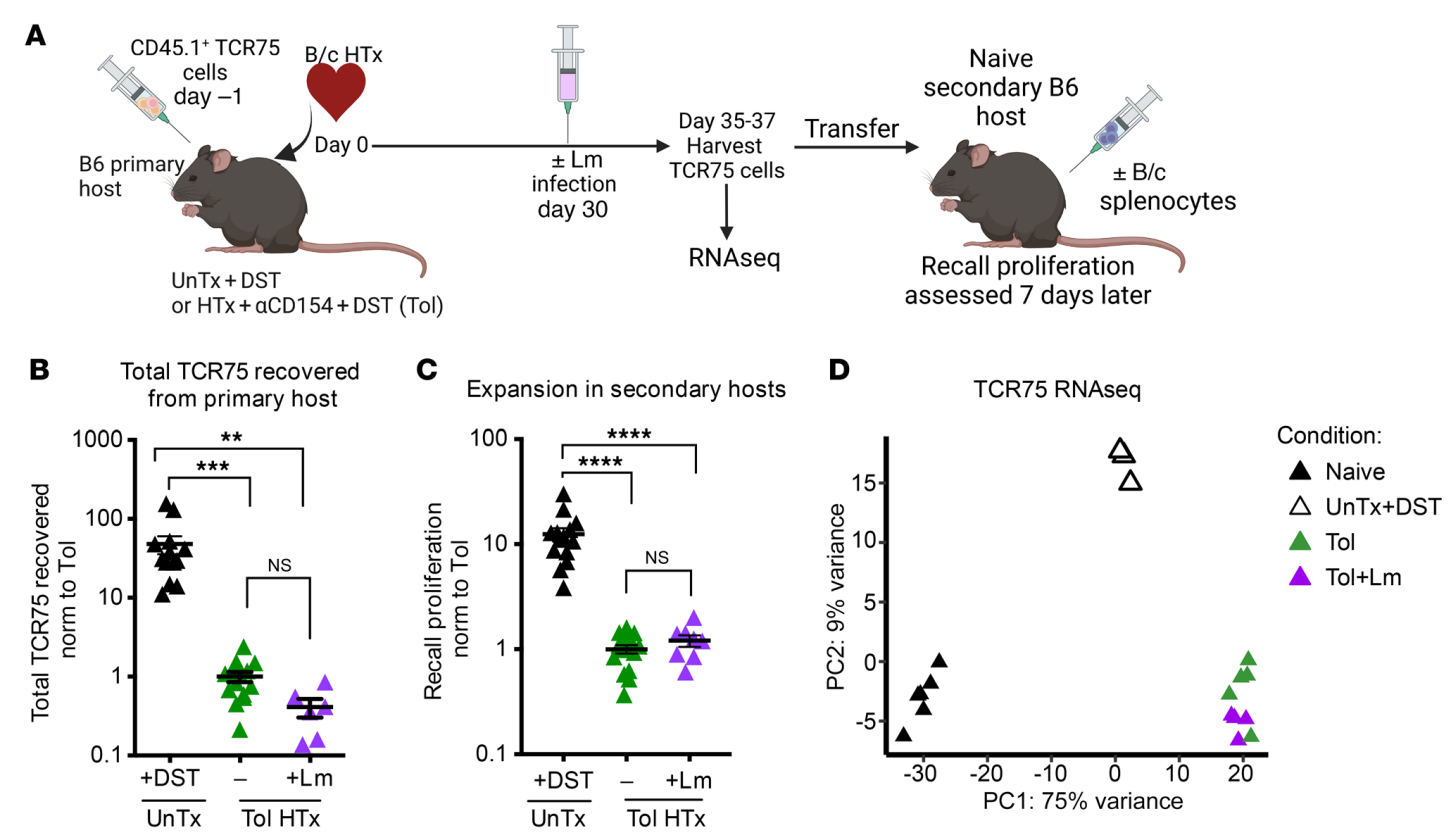
Donor MHCI–specific T cells remain hypofunctional following Lm infection. (**A**) Experimental design. CD4^+^CD45.1^+^ TCR75/*Rag^–/–^* T cells (TCR75) were adoptively transferred into CD45.2^+^ B6 hosts either untransplanted and immunized with B/c donor splenocytes i.p. (UnTx+DST) to induce memory or prior to transplantation of a B/c heart (HTx) in the presence of αCD154 (day 0, day 7, day 14) and DST (day 0) to induce tolerance (Tol). A group of tolerant mice were infected with Lm on day 30 after transplantation (Tol+Lm). CD45.1^+^ TCR75 cells were harvested from the spleen and lymph nodes on days 35–37 after transplantation in all groups, enumerated, and subjected to bulk RNA-Seq. For recall proliferation, an equal number of harvested, sorted TCR75 cells were adoptively transferred into new congenic naive B6 hosts immunized with B/c splenocytes. Cells were enumerated from the spleen 7 days after in vivo rechallenge. (**B**) Fold change of TCR75 cells recovered from UnTx+DST (*n* = 13), Tol (*n* = 14), and Tol+Lm (*n* = 6, day 7 after Lm) mice, normalized to the number recovered in uninfected Tol animals. (**C**) Expansion in secondary hosts. Fold change of total TCR75 cells recovered from spleens of secondary hosts normalized to the cells recovered when TCR75 cells originated from Tol hosts prior to adoptive transfer. UnTx+DST (*n* = 14), Tol (*n* = 15), Tol+Lm (*n* = 8). (**D**) Principal component (PC) analysis of RNA-Seq. Gene expression comparison between naive TCR75 cells (day 0, *n* = 6), and memory TCR75 (UnTx+DST day 35, *n* = 3) or tolerant TCR75 cells from uninfected (Tol day 35, *n* = 5) or Tol+Lm analyzed day 5 after infection (*n* = 4). Statistical comparisons were performed with 1-way ANOVA with Bonferroni’s correction for multiple pairwise comparisons. **P < 0.01, ***P < 0.001, ****P < 0.0001.

Lm-mediated cardiac allograft rejection following establishment of transplantation tolerance was T cell dependent and correlated with transiently detectable alloreactivity (5, 13), although the T cells responsible for this IFN-γ production in response to donor alloantigen stimulation remained to be identified. We had previously shown that alloreactive CD4^+^ TCR75 cells, which are specific for a donor MHCI–derived peptide presented indirectly on host MHCII, became hypofunctional in tolerant mice and did not show recovered function when tested at day 30 after Lm infection of tolerant hosts (12). It remained possible that these T cells were reinvigorated early following infection, but then returned to their hypofunctional phenotype over time following Lm clearance. Alternatively, alloreactive T cells of a differing specificity might retain function despite the tolerance induction regimen and be responsible for Lm-dependent graft loss. To distinguish between these possibilities, we first assessed whether TCR75 cells regained function early following Lm infection. TCR75 cells (CD45.1^+^ and on a *Rag^–/–^* background) were adoptively transferred into congenic C57BL/6 (B6) recipients (CD45.2^+^) 1 day before transplantation with a BALB/c (B/c) heart and induction of tolerance with αCD154+DST (CoB). Thirty days after transplantation, some recipient mice were infected with Lm, and TCR75 cell function was assessed 7 days after infection (Figure 1A). As controls, TCR75 cells were injected into B6 mice 1 day prior to immunization with B/c DST to generate memory TCR75 cells, and these cells were harvested 37 days later. TCR75 cell numbers recovered from the tolerant hosts were lower than those from DST-immunized hosts, reflecting the abortive proliferation resulting from the αCD154 treatment (Figure 1B). Similarly to our prior findings when TCR75 cells were analyzed at 30 days after Lm infection of tolerant hosts (12), cell numbers of TCR75 cells at 7 days after Lm infection of tolerant hosts were not greater than those in uninfected tolerant hosts (Figure 1B). Additionally, TCR75 cells from infected tolerant mice did not regain the ability to proliferate upon donor splenocyte rechallenge in naive secondary hosts, away from the tolerant environment of the primary host (Figure 1C), substantiating a lack of refunctionalization after infection. We further compared gene expression by bulk RNA-Seq among TCR75 cells from naive mice, memory TCR75 cells from mice immunized with B/c splenocytes, and TCR75 cells from tolerant mice, either uninfected or infected with Lm 5 days prior. TCR75 cells from tolerant and Lm-infected mice clustered tightly together by principal component analysis (PCA) and displayed extremely similar gene-expression profiles (Figure 1D), with only 10 genes differentially expressed (not shown). It thus appeared unlikely that Lm-mediated rejection, which occurs on days 10 to 20 after infection (5), results from reinvigoration of chronically stimulated endogenous Tconvs that acquire a hypofunctional phenotype similar to that of TCR75 cells.

### Presentation of donor MHCII–derived peptide decreases with time.

**Figure 2:**
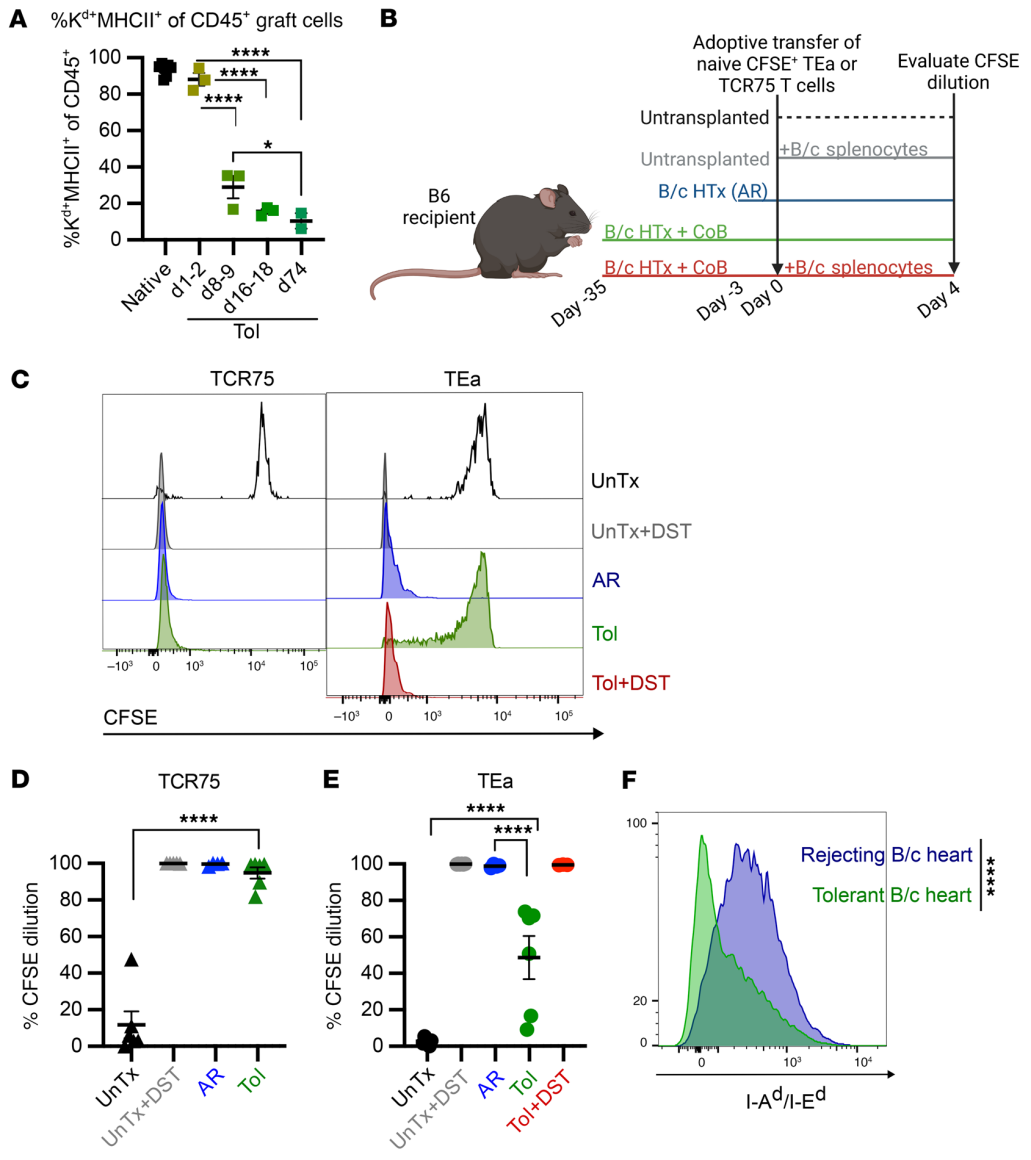
The presentation of donor MHCII–derived peptide declines during tolerance. (**A**) Cardiac allograft infiltrating CD45^+^ hematopoietic cells expressing MHCII. Native represents B/c hearts directly taken ex vivo. Grafts from Tol mice were analyzed between days 1 and 74 after transplantation. Native (*n* = 7), days 1–2 (*n* = 3), days 8–9 (*n* = 3), days 16–18 (*n* = 3), day 74 (*n* = 2). Significance not pictured: native versus days 1–2 (****), native versus days 8–9 (****), native versus days 16–18 (****), native versus days 74 (****). *P < 0.05, ****P < 0.0001. (**B**) Experimental design for **C** and **E**. Mice were transplanted with a B/c heart, and tolerance was induced with αCD154/DST (CoB). Thirty-five days after transplantation, CFSE-labeled CD45.1^+^TCR75/*Rag^–/–^* (TCR75) or CD45.1^+^TEa/*Rag^–/–^* (TEa) cells were adoptively transferred (Tol). Some control mice received a B/c heart without CoB 3 days prior to TCR-Tg cell transfer (AR). Other control mice were immunized with B/c splenocytes the same day as the TCR-Tg adoptive transfer (UnTx+DST). One subset of tolerant mice received extra alloantigen in the form of B/c splenocytes on the day of TEa transfer (Tol+DST). In all cases, TCR-Tg T cells were recovered 4 days after adoptive transfer and evaluated for CFSE dilution. (**C**) Representative histograms showing CFSE dilution in TCR75 or TEa cells 4 days after adoptive transfer into the groups described in **B**. (**D** and **E**) Summary data of CFSE dilution in TCR75 (**D**) or TEa (**E**) cells 4 days after adoptive transfer into the groups described in **B**. Data are represented as mean ± SEM. For **D**, UnTx (*n* = 6), UnTx+DST (*n* = 5), AR (*n* = 6), Tol (*n* = 6). For **E**, UnTx (*n* = 6), UnTx+DST (*n* = 4), AR (*n* = 5), Tol (*n* = 6), Tol+DST (*n* = 3). Significance not pictured: UnTx versus UnTx+DST (****), UnTx+DST versus Tol (****), Tol versus Tol+DST (***). (**F**) Representative histograms of donor MHCII (I-A^d^/I-E^d^) expression on graft-derived CD45^–^CD31^+^H2K^d+^ endothelial cells. Data are representative of summary data shown in Figure 5B. Data were compared by 1-way ANOVA with Bonferroni’s correction for multiple pairwise comparisons (**A**, **D**, and **E**) or unpaired 2-tailed *t* test (**F**). *P* < 0.05 was considered significant. ***P < 0.001, ****P < 0.0001.

In mice transplanted with fully MHC mismatched heart allografts, it has been shown that host T cells specific for donor MHCI or their derived peptides have the capacity to engage alloantigen long term, as donor MHCI is persistently expressed by most graft cells (15). In contrast, responses to donor MHCII–derived antigens are more transient (15), as passenger APCs, a major source of donor MHCII, die shortly after transplantation (Figure 2A). Graft endothelial cells can upregulate expression of MHCII in response to IFN-γ (17, 18), but endothelial cells may not provide sufficient donor MHCII to persistently stimulate T cells. To assess the potential for donor MHCI– or MHCII–derived alloantigens to activate allospecific T cells during the maintenance phase of tolerance, we adoptively transferred CFSE-labeled naive TCR-Tg T cells more than 30 days after B/c heart transplantation into B6 recipients that had been treated at the time of transplantation with CoB to induce allograft tolerance. CFSE dilution was assessed 4 days after naive TCR-Tg cell transfer as a readout of cell proliferation in response to alloantigen expressed by the host (Figure 2B). To detect the presence of donor MHCI and MHCII, we used TCR75 TCR-Tg cells and TEa TCR-Tg cells (both CD45.1^+^ on a *Rag^–/–^* background) that recognize a donor K^d^–derived peptide and a donor I-E^d^–derived peptide, respectively, both presented indirectly on host I-A^b^. As expected, almost all naive CFSE-labeled TCR75 cells proliferated sufficiently to fully dilute CFSE (Figure 2, C and D), reflecting the persistence of donor MHCI expression in the graft of tolerant hosts. Conversely, CFSE-labeled TEa cells displayed significantly reduced proliferation after adoptive transfer into similarly tolerant mice (Figure 2, C and E), suggesting lower expression and/or presentation of donor MHCII–derived peptide in the tolerant hosts at the maintenance phase of tolerance. Importantly, TEa cells proliferated to an extent similar to that of TCR75 cells after transfer into naive B6 mice immunized with B/c splenocytes, indicating that naive TEa cells can proliferate in the presence of sufficient alloantigen. It was also possible that even fresh heart allografts do not contain sufficient MHCII to stimulate TEa cell proliferation. This was not the case, as both TEa and TCR75 cells fully diluted CFSE when transferred into nonimmunosuppressed mice transplanted with a B/c heart 3 days prior and undergoing acute rejection (AR) (Figure 2, C–E). Indeed, proliferation of TEa cells in mice undergoing heart allograft rejection correlated with higher expression of donor MHCII on graft endothelial cells when compared with expression on endothelial cells from established tolerant grafts (Figure 2F). Finally, provision of additional antigen in the form of B/c splenocytes could rescue naive TEa proliferation after transfer into stably tolerant heart recipients (Figure 2, C and E), suggesting that the limited TEa response to heart allografts at the maintenance phase of tolerance was due to low donor MHCII–derived peptide antigen availability and not to dominant suppression of these cells. Overall, antigen was limiting for TEa cells, but not for TCR75 cells, in tolerant mice.

### T cells specific for donor MHCII–derived peptide retain more functionality than T cells specific for donor MHCI–derived peptide during the maintenance phase of tolerance.

**Figure 3:**
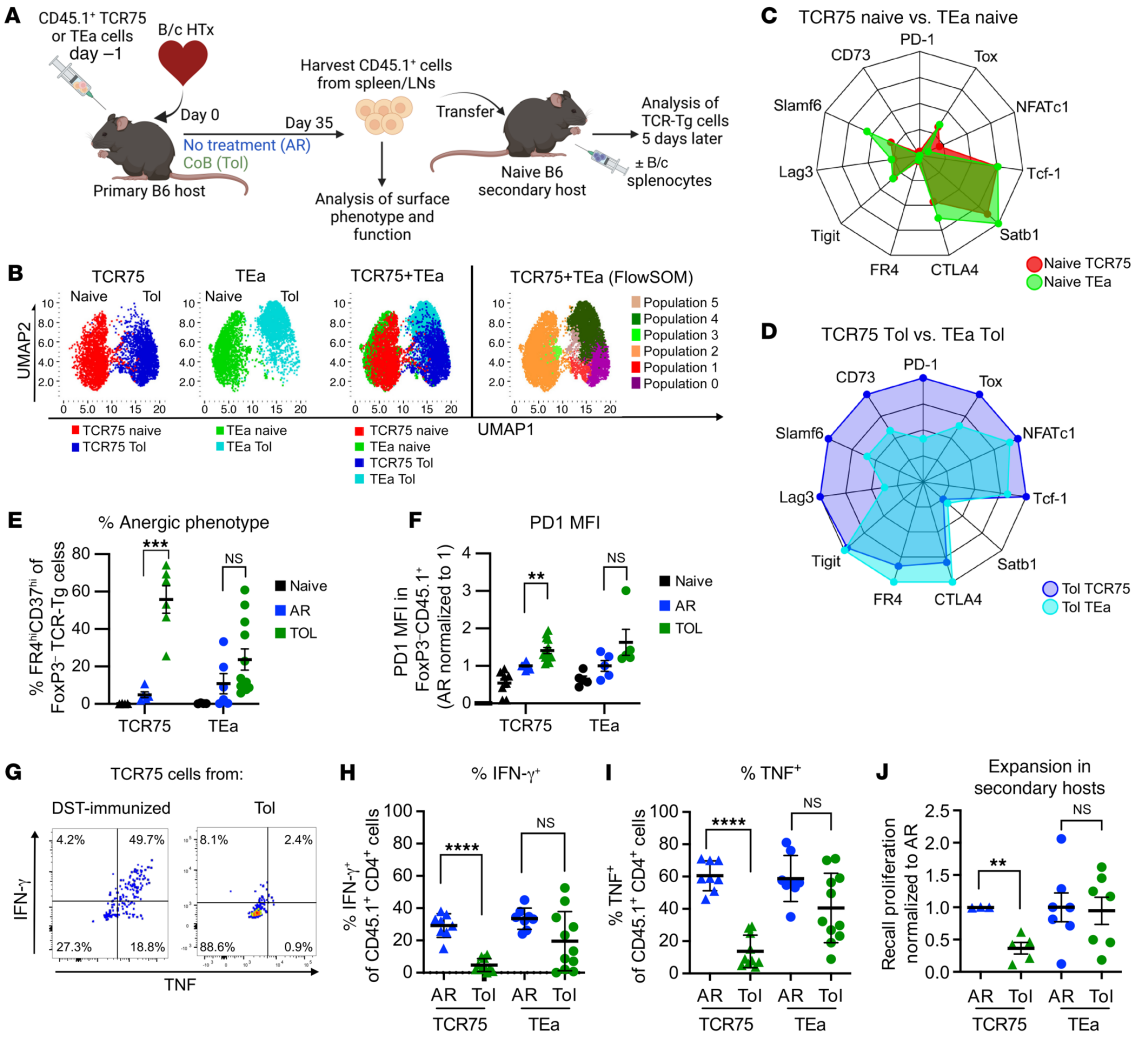
T cells specific for donor MHCII retain function following induction of transplantation tolerance. (**A**) Experimental design. (**B**, **C**, and **D**) UMAP plots (**B**) generated from 2,000 live TCR-Tg Tconvs per condition. TCR75 (left) and TEa (center) cells shown separately and together (right). FlowSOM (far right) identified distinct clusters, distinguishing between naive (populations 2 and 3), Tol TCR75 cells (populations 0, 1, 5), and Tol TEa (population 4) cells. Radar plots showing the relative expression of markers (as percentage of maximal expression) between naive (**C**) or Tol (**D**) TCR-Tg cells. *n* = 3–4 per group. (**E** and **F**) Percentage of TCR-Tg Tconvs expressing CD73^hi^FR4^hi^ (**E**) or MFI of PD1 (**F**). Results were pooled from 2–4 independent experiments. **E**: Naive TCR75 (*n* = 4), AR TCR75 (*n* = 5), Tol TCR75 (*n* = 6), naive TEa (*n* = 5), AR TEa (*n* = 6), Tol TEa (*n* = 12). **F**: naive TCR75 (*n* = 8), AR TCR75 (*n* = 11), Tol TCR75 (*n* = 14), naive TEa (*n* = 5), AR TEa (*n* = 5), Tol TEa (*n* = 5). Data are represented as mean ± SEM. Significance not depicted in panel **E**: naive TCR75 versus Tol TCR75 (****), naive TEa vs Tol TEa (*). Significance not depicted in panel **F**: naive TCR75 versus Tol TCR75 (**), naive TEa versus Tol TEa (*), naive TEa versus AR TEa (**). (**G**) Representative flow plots. TCR75 cells were seeded at the time of B/c splenocyte immunization (DST) or B/c heart transplantation+CoB treatment (Tol). Splenocytes were harvested on day >30 and plated overnight with anti-CD3/CD28. CD4^+^CD45.1^+^ gated events were analyzed for the percentage of IFN-γ^+^ and/or TNF^+^ cells. (**H** and **I**) Percentages of TCR-Tg T IFN-γ (**H**) or TNF (**I**) post anti-CD3/CD28 restimulation. Results were pooled from 3 independent experiments. Each data point represents a sample pooled from 1–5 mice (mean ± SEM). AR TCR75 (*n* = 8), Tol TCR75 (*n* = 10), AR TEa (*n* = 8), Tol TEa (*n* = 11). (**J**) Splenic TCR-Tg T cells enumerated 5 days post-DST immunization of secondary hosts. Results normalized to the average cell recovery of TCR-Tg T cells originating from AR primary hosts, set to 1 for each independent experiment (mean ± SEM). AR TCR75 (*n* = 3), Tol TCR75 (*n* = 5), AR TEa (*n* = 7), Tol TEa (*n* = 7). Data were analyzed by 1-way ANOVA with Bonferroni’s correction for multiple pairwise comparisons (**E** and **F**). Data comparing TCR75 or TEa cells in AR versus Tol (**H**–**J**) were analyzed by unpaired 2-tailed *t* test. *P < 0.05, **P < 0.01, ***P < 0.001, ****P < 0.0001 for all data

Donor MHCII expression during tolerance was insufficient to stimulate full proliferation of naive TEa cells (Figure 2, C and E), and we knew that persistent antigen stimulation was required for acquisition of hypofunction in TCR75 cells exposed to CoB therapy at the induction of tolerance (12). Thus, we hypothesized that insufficient chronic stimulation may allow TEa cells to retain functionality during tolerance induction (16). To test this hypothesis, we compared the phenotype and function of tracer TCR75 versus tracer TEa cells, seeded at the time of transplantation into untreated or CoB-treated recipients of B/c hearts. TCR-Tg cells were isolated 35 or more days after transplantation and T cell transfer (Figure 3A), and the phenotype of CD44^hi^ FoxP3^–^ Tconvs was analyzed by spectral flow using a large panel of antibodies to surface markers and transcription factors associated with exhaustion. UMAP (https://umap-learn.readthedocs.io/en/latest/) (Figure 3B) and radar plots (Figure 3C) revealed quasi-complete overlap in the phenotype of naive TCR75 and naive TEa cells. In contrast, in tolerant mice, the phenotype of TCR75 cells was markedly distinct from that of TEa cells, a result confirmed by FlowSOM analysis (https://bioconductor.org/packages/release/bioc/html/FlowSOM.html) (Figure 3B) when TCR75 and TEa cells were analyzed together. FlowSOM revealed 1 major population in the naive groups (orange population 2 corresponding to both naive TCR75 and naive TEa) and 2 distinct major populations in the tolerant groups (dark green population 4 corresponding preferentially to Tol TEa, and pink population 0 corresponding preferentially to Tol TCR75). Most notably, in tolerant hosts, TCR75 cells expressed greater levels than TEa cells of the markers of activation and exhaustion PD-1, Lag3, and Slamf6 (19, 20), of the anergy marker CD73 (21), and of the transcription factor associated with exhaustion Tox (22–24) (Figure 3D), all consistent with a more dysfunctional “exhausted” phenotype of TCR75 cells when compared with TEa cells. Higher percentages of double expressors of the anergy markers FR4 and CD73 (21) and greater levels of PD-1 expression in TCR75 than TEa cells from tolerant mice were confirmed by conventional flow cytometry, with the percentages of FR4^hi^CD73^hi^ on TEa cells being more variable and not significantly different in tolerant than in rejected mice (Figure 3E). Similarly, expression of PD-1 was significantly higher on TCR75 cells from tolerant than rejected mice, but not on TEa cells (Figure 3F). These results indicate that the anergy/exhausted phenotype was more variable and less established in TEa than TCR75 cells from tolerant mice.

To investigate function, TEa and TCR75 cells isolated from tolerant and rejected heart allograft recipients 35 days after transplantation were subjected to restimulation in vitro or in vivo (Figure 3A). Figure 3G shows a representative plot of cytokine production upon in vitro restimulation with anti-CD3/CD28, by memory TCR75 cells at day 35 after DST immunization and loss of cytokine production by TCR75 cells from transplanted tolerant mice. As memory cells, both TCR75 and TEa cells from rejected mice were able to produce IFN-γ and TNF upon in vitro restimulation (Figure 3, H and I). TCR75 cells from tolerant mice were significantly impaired in their production of both IFN-γ and TNF (Figure 3H). In contrast, TEa cells from tolerant mice retained IFN-γ and TNF production comparable to that of TEa cells from acutely rejected mice (Figure 3, H and I). Furthermore, in the recall proliferation assay in secondary hosts restimulated with B/c splenocytes in vivo, TEa cells from tolerant mice accumulated similarly to TEa cells from rejected mice, whereas we confirmed the impaired recall proliferation of TCR75 cells from tolerant mice (Figure 3J). These data demonstrate that, unlike TCR75 cells, TEa cells retain functionality in tolerant hosts, raising the possibility that T cells specific for alloantigens whose expression decreases over time retain more function following tolerance induction than allospecific T cells recognizing persistently expressed antigens.

Alternatively, differences in functionality between TEa and TCR75 cells from tolerant hosts may reflect differences in TCR-Tg–intrinsic properties. To address this potential confounder, we used T cells of a single specificity, OVA-reactive OTII TCR-Tg CD4^+^ T cells (also on a *Rag^–/–^* and CD45.1^+^ background), and we varied the duration of expression of their cognate antigen within the graft. To this end, we obtained TGO mice as transplant donors in which temporal expression of the antigen OVA can be controlled by ingestion of a doxycycline-containing (Dox-containing) diet (25). These mice were crossed to M2-rtTA–expressing mice to ensure all tissues could express OVA (Supplemental Figure 1A; supplemental material available online with this article; https://doi.org/10.1172/JCI168465DS1), allowing us to control duration of OVA expression in the heart (Supplemental Figure 1, B and C) and skin (Supplemental Figure 2) transplants. We confirmed control of OVA expression upon administration (Supplemental Figure 1B and Supplemental Figure 2A) or cessation (Supplemental Figure 2C) of Dox chow by evaluating CFSE dilution of OTII TCR-Tg CD4^+^ T cells. Two days of Dox chow prior to OTII transfer were sufficient to drive OTII proliferation (Supplemental Figure 1C and Supplemental Figure 2B), whereas cessation of Dox chow 3 days prior to OTII transfer was sufficient to extinguish OVA expression, preventing proliferation of OTII cells (Supplemental Figure 2D).

**Figure 4:**
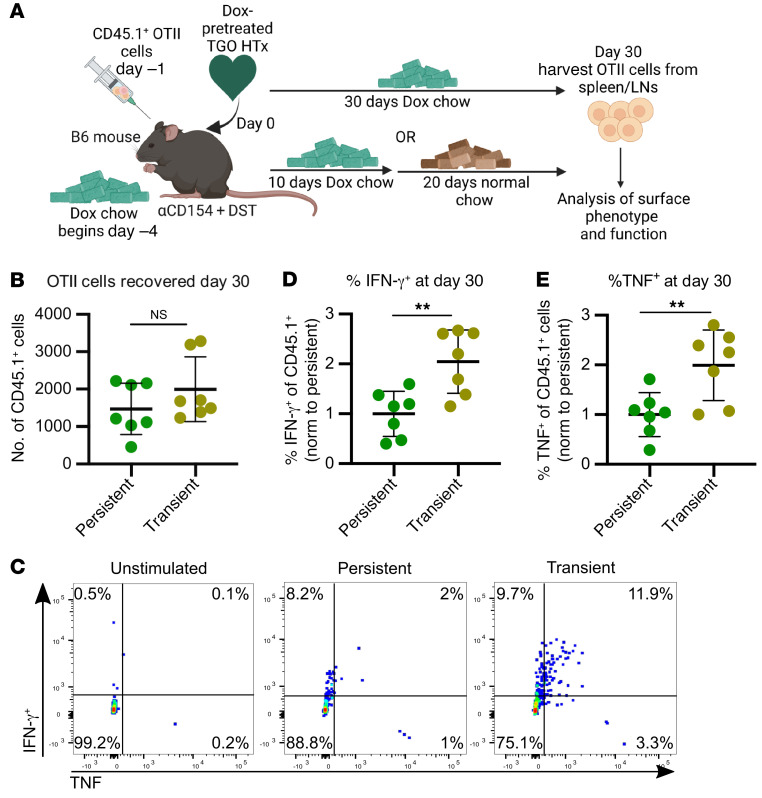
Persistent expression of cognate antigen on donor grafts of tolerant mice results in greater OTII cell hypofunction than transient antigen expression. (**A**) Experimental design. CD45.2^+^ B6 recipients were pretreated with a Dox-containing diet 4 days prior to transplantation. Recipients were also adoptively transferred with CD45.1^+^ OTII cells (on a *Rag^–/–^* background) 1 day prior to transplantation with TGO hearts harvested from Dox chow–fed donors. All hosts were treated with CoB. After transplantation, mice were maintained on a Dox diet for either 30 days (Persistent) or only 10 days, returning to a normal chow for the remaining 20 days of the experiment (Transient). (**B**) Total CD4^+^CD45.1^+^ OTII cells recovered from spleens and lymph nodes on day 30 or later from mice described in **A**. Each data point represents a sample from 1 mouse with lines indicating mean ± SEM. Persistent OVA (*n* = 7), transient OVA (*n* = 7). (**C**) Representative flow plots of cytokine expression. B6 mice were prepared as described in **A**. Spleens and lymph nodes were harvested on day 30 or later, and a fraction of cells were restimulated in vitro overnight with anti-CD3/CD28 in the presence of brefeldin A, with some cells remaining unstimulated and exposed to brefeldin A as controls. CD4^+^CD45.1^+^ gated events were analyzed for the percentage of cells producing IFN-γ and/or TNF. (**D** and **E**) Percentages of OTII cells producing IFN-γ or TNF normalized to the average percentage in the Persistent OVA group in each independent experiment. Results were pooled from 2 independent experiments. Each data point represents a sample from 1 mouse, with lines indicating mean ± SEM. Persistent OVA (*n* = 7), Transient OVA (*n* = 7). **P < 0.01. Data were compared using unpaired 2-tailed *t* test.

To determine whether durable versus transient expression of the same cognate antigen on an allograft would ensure OTII hypofunction versus retained functionality, OTII cells were transferred into congenic B6 hosts 1 day prior to transplantation with TGO hearts from donors on a Dox chow. All hosts were treated with CoB at the time of transplantation. Hosts received Dox chow from the day of transplantation for either 10 days (transient alloantigen exposure) or 30 days (persistent alloantigen exposure), and all animals were analyzed at 30 days after transplantation (Figure 4A). As a result of CoB treatment, there was no difference in the number of CD45.1^+^ OTII cells recovered following transient or persistent alloantigen exposure (Figure 4B). Upon overnight in vitro restimulation, OTII cells exposed to persistent Dox chow displayed reduced production of IFN-γ and TNF compared with OTII cells from mice exposed to transient Dox chow (Figure 4, C–E). These findings are consistent with our observations of greater loss of function in TCR75 than TEa cells in tolerant hosts and support that hypofunction of allospecific CD4^+^ T cells following a tolerogenic regimen depends on persistent expression of the cognate antigen.

### Lm infection at the maintenance phase of tolerance upregulates donor MHCII expression and induces TEa expansion.

**Figure 5:**
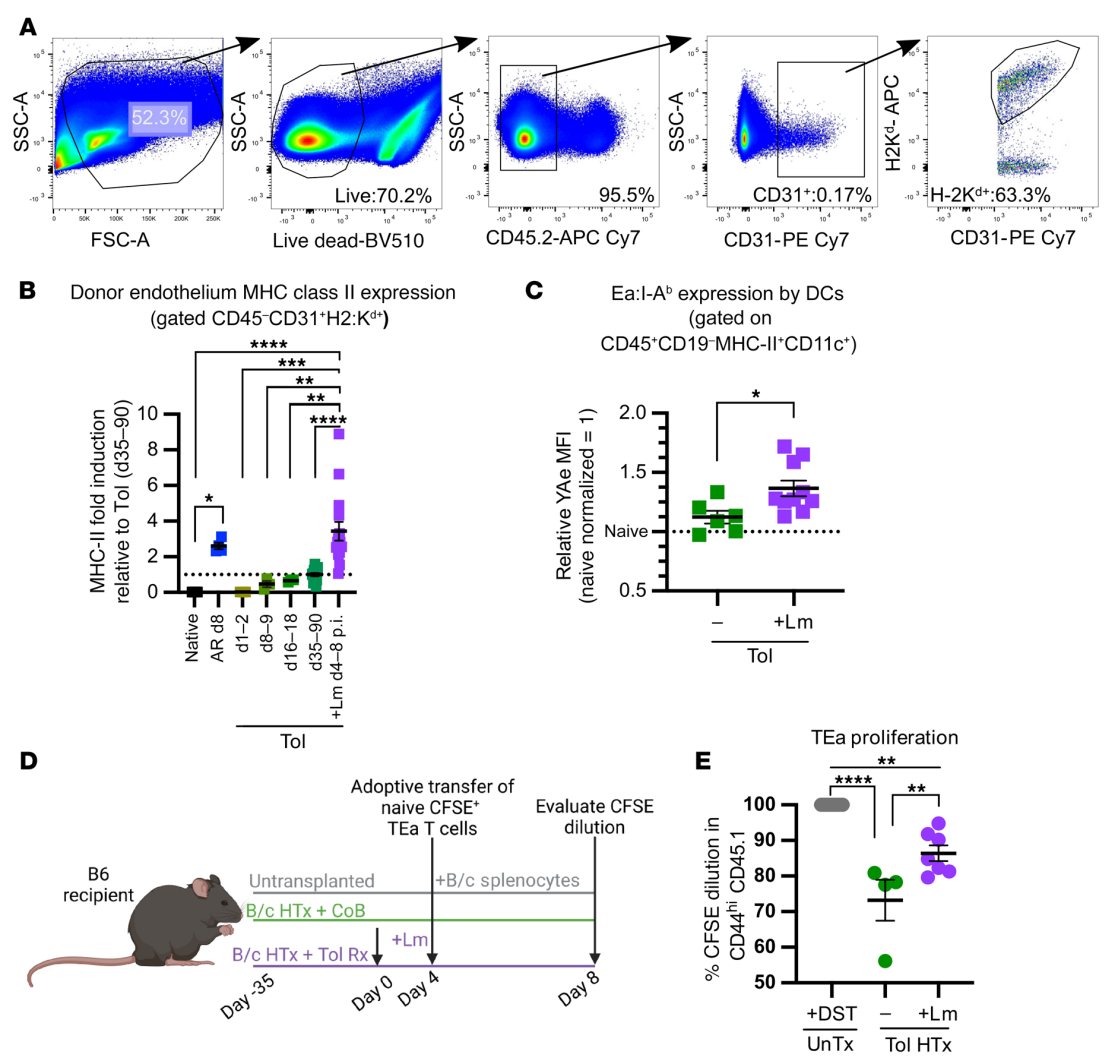
Increased presentation of donor MHCII–derived peptides can be induced in settings of Lm infection at the maintenance phase of tolerance. (**A**) Gating strategy for endothelial cells from heart allografts. (**B**) I-A^d^/I-E^d^ expression on graft-isolated CD45^–^CD31^+^K^d+^ endothelial cells. Native represents B/c hearts directly taken ex vivo. AR represents hearts from untreated mice at day 8 after transplantation. Tol grafts were analyzed between days 1 and 90 after transplantation+CoB. Tol+Lm mice were infected day 30 after transplantation+CoB, and grafts were evaluated on days 4–8 after infection. Native (*n* = 8), AR (*n* = 4), days 1–2 (*n* = 3), days 8–9 (*n* = 3), days 16–18 (*n* = 3), days 35–90 (*n* = 16), Tol+Lm d-8 p.i. (*n* = 15). For comparison between groups, results were normalized to levels of expression of MHCII in endothelial cells from tolerant grafts at days 35–90 after transplantation (dotted line). (**C**) B6 mice transplanted with B/c hearts and treated with CoB were infected or not with Lm on day 30 after transplantation. Splenocytes were analyzed 4–8 days later for expression of Eα:I-A^b^ on CD11c^+^ gated events and normalized to the average expression on DCs from naive mice in each independent experiment. Tol (*n* = 6), Tol+Lm (*n* = 9). (**D**) Experimental model. Some B6 mice received a B/c heart graft, and tolerance was induced with CoB (Tol HTx). Thirty-five days after transplantation, a subset of these mice were infected with Lm (Tol HTx+Lm). Four days after infection, all mice were adoptively transferred with CFSE-labeled naive TEa cells. Control untransplanted mice received TEa cells at the same time as B/c splenocyte immunization (UnTx+DST). All TEa cells were recovered on day 4 after adoptive transfer. (**E**) Percentages of CD45.1^+^ TEa cells that proliferated on day 4 after adoptive transfer. UnTx+DST (*n* = 5), Tol (*n* = 4), Tol+Lm (*n* = 7). Data were analyzed by 1-way ANOVA with Bonferroni’s correction for multiple pairwise comparisons (**B** and **E**) or unpaired 2-tailed *t* test (**C**). *P < 0.05, **P < 0.01, ***P < 0.001, ****P < 0.0001.

Alloreactive T cells specific for transiently expressed alloantigens that retain functionality following tolerance induction may pose a threat to the graft if their cognate antigen becomes reexpressed. Although levels of donor MHCII–derived alloantigen were insufficient to fully stimulate the proliferation of naive TEa cells transferred at the maintenance phase of transplantation tolerance (Figure 2, C and E), an inflammatory event leading to an increase in IFN-γ production, such as Lm infection, might be able to trigger upregulation of donor MHCII on the previously tolerated allograft (17, 18). To determine whether Lm infection results in upregulation of donor MHCII on endothelial cells of tolerant grafts, tolerant heart recipients were infected with Lm at day 30+ after transplantation with CoB treatment and CD45^–^CD31^+^ donor-derived endothelial cells from the graft were evaluated 4 to 8 days after infection for expression of I-A^d^/I-E^d^ (gating shown in Figure 5A). When compared with donor endothelial cells from uninfected tolerant recipients at various time points after transplantation, Lm infection led to a marked increase in I-A^d^/I-E^d^ expression, with donor MHCII levels similar to those in donor endothelial cells from actively rejecting allografts analyzed on day 8 after transplantation (Figure 5B). Moreover, host splenic DCs displayed increased presentation of donor MHCII–derived peptide (Eα) presented on I-A^b^ at days 4 to 8 after Lm infection of tolerant hosts, as detected by staining with the YAe antibody (Figure 5C). To verify whether Lm infection–dependent upregulation of donor-derived MHCII was sufficient to be detected by donor MHCII peptide–reactive T cells, naive TEa cells were CFSE labeled and adoptively transferred into tolerant heart-graft recipients 4 days after Lm infection (Figure 5D). Indeed, these naive TEa cells experienced significantly greater CFSE dilution when transferred into Lm-infected than uninfected tolerant mice (Figure 5E). Together, these data suggest that host T cells recognizing donor MHCII indirectly have the potential to be reactivated by their cognate alloantigen during an infection.

**Figure 6:**
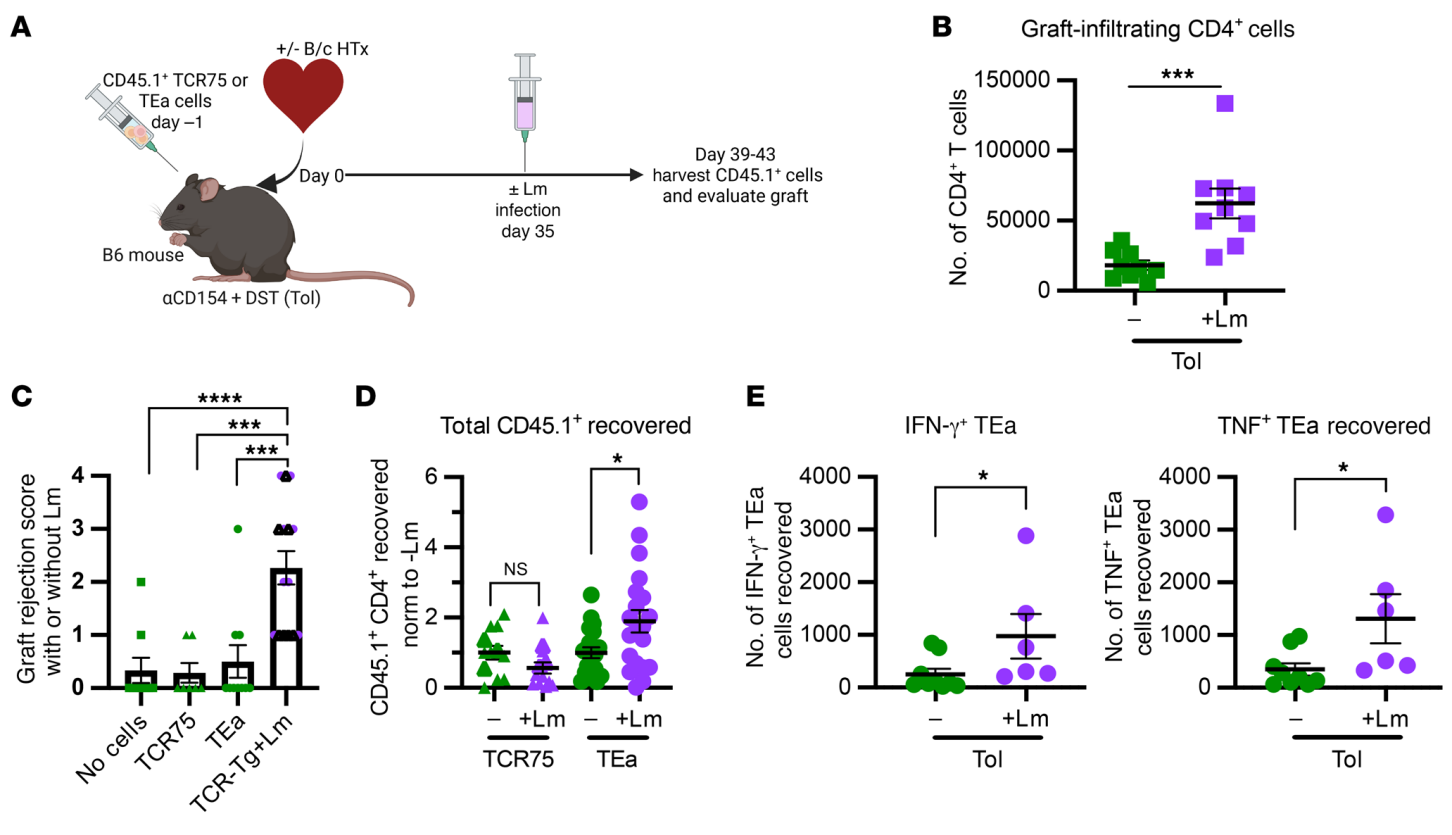
Lm infection in tolerant hosts induces upregulation of MHCII on donor endothelium and results in expansion of cytokine-producing TEa cells. (**A**) Experimental design for **D**–**E**. TCR75 or TEa cells were adoptively transferred into congenic B6 recipients prior to transplantation with a B/c heart allograft and treatment with CoB to induce tolerance in all hosts. After 35+ days, a subset of mice were infected with Lm. Four to 8 days after infection, heart grafts were palpated and CD45.1^+^ T cells were recovered, counted, and analyzed by flow cytometry. (**B**) Total CD4^+^ T cells recovered from transplanted hearts at days 39 to 43 after transplantation. Tol (*n* = 9); Tol+Lm (*n* = 9). (**C**) Heart graft palpation score days 39 to 43. Hearts were scored, with a perfect score as 0 and a rejected heart as 4, on the following criteria: presence of heartbeat (absent = 1 point), graft size (enlarged =1 point), heartbeat speed (slow = 1 point), strength of heartbeat (weak = 1 point). No cells (*n* = 9), TCR75 (*n* = 7), TEa (*n* = 10), TCR-Tg (either TCR75 or TEa) +Lm (*n* = 15). (**D**) Total CD45.1^+^ T cells recovered from spleen and lymph nodes at days 39–43 after transplantation. TCR75 (*n* = 12), TCR75+Lm (*n* = 13), TEa (*n* = 20), TEa+Lm (*n* = 20). (**E**) The total number of CD45.1^+^ TEa T cells recovered by FACS was multiplied to the percentage of cells producing cytokines upon restimulation with anti-CD3/CD28 in vitro in the presence of brefeldin A. Values represent the number of cytokine-producing CD45.1^+^ TEa T cells per mouse. Tol (*n* = 9), Tol+Lm (*n* = 6). All data points represent a sample pooled from 1–2 mice, with lines indicating mean ± SEM. Data were analyzed by 2-tailed unpaired *t* test (**B**, **D**, and **E** comparing ± Lm for each TCR-Tg group) or 1-way ANOVA with Bonferroni’s correction for multiple pairwise comparisons (**C**). *P < 0.05, ***P < 0.001, ****P < 0.0001

To determine whether T cells that have been subjected to tolerance induction become reactivated following infection, we adoptively transferred TCR75 or TEa cells at the time of transplantation and CoB treatment. Tolerant heart-graft recipients were infected with Lm at day 30+ after transplantation, and the congenic T cells were recovered between days 4 and 8 after infection (Figure 6A). Following infection, we saw an increase in the number of CD4^+^ T cells infiltrating the graft (Figure 6B). Adoptive transfer of TEa or TCR75 cells did not affect the induction of tolerance, as revealed by similar graft rejection scores in mice with or without transferred T cells, and Lm infection worsened the graft rejection score similarly (Figure 6C), in keeping with our previous studies demonstrating that Lm can break established tolerance (5). Consistent with the increased expression of donor MHCII induced by Lm infection of graft endothelial cells (Figure 5B), more TEa cells were recovered from infected than uninfected hosts 1 week after infection, whereas Lm infection had no impact on the number of TCR75 cells recovered (Figure 6D). While the percentage of TEa cells producing cytokines (Supplemental Figure 3, A and B) and the amount of cytokine produced on a per-cell basis (MFI) (Supplemental Figure 3, C and D) were similar before and after Lm infection, the expansion of TEa cells after Lm infection resulted in an increase in the total number of cytokine-producing TEa T cells (Figure 6E). Thus, T cells specific for donor MHCII–derived peptide but not donor MHCI–derived peptide significantly (*P* < 0.05) expand following Lm infection of tolerant hosts. Combined with their maintained ability to produce cytokines, TEa cells, and presumably endogenous T cells of both direct and indirect specificities to donor MHCII or to polymorphic stress/inflammation-induced proteins, may represent allospecific T cells that participate in allograft rejection during Lm infection of tolerant hosts.

### Repeated injections of donor splenocytes widen the repertoire of allospecific T cells that are hypofunctional in CoB-treated mice and confer resistance to Lm-dependent rejection.

**Figure 7:**
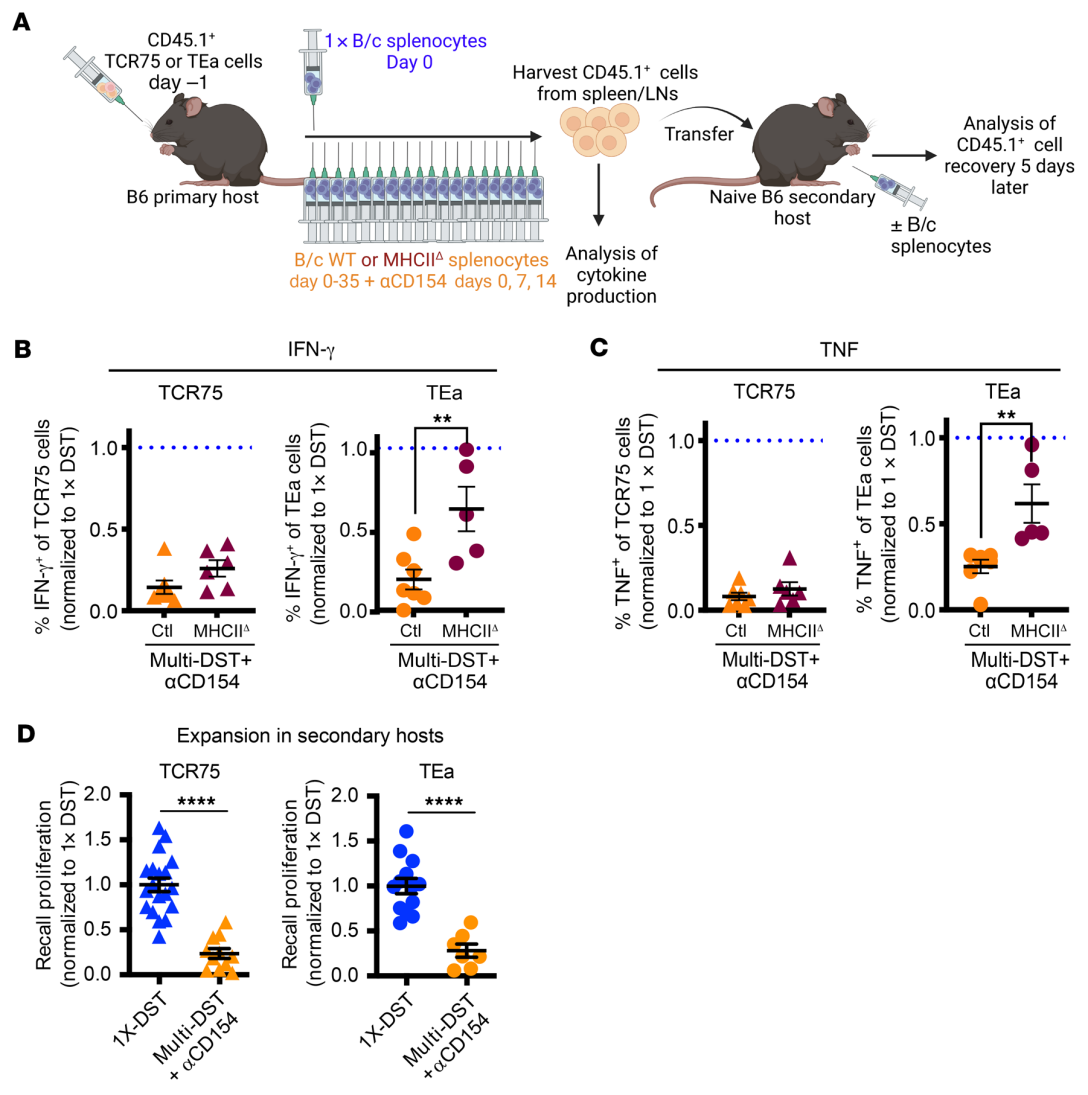
Donor MHCI– and MHCII–reactive T cells become dysfunctional with prolonged exposure to their cognate alloantigen. (**A**) Experimental design. B6 mice were adoptively transferred with TCR75 or TEa cells, then either immunized with a single injection of B/c splenocytes (1X-DST) or given αCD154 (days 0, 7, and 14) and repeated injections of either B/c splenocytes (Ctl) or B/c splenocytes depleted of MHCII^+^ cells (MHCII^Δ^, except the first of the 18 injections used Ctl B/c splenocytes to activate TEa cells) every 48 hours until sacrifice on day 35 (multi-DST+αCD154). (**B** and **C**) Percentages of TCR-Tg cells producing IFN-γ (**B**) and TNF (**C**) following cell isolation from spleen+lymph nodes of primary hosts on day 35 and restimulation overnight with anti-CD3/CD28 in the presence of brefeldin A. For TCR75 cells, 1×-DST (*n* = 11), multi-DST+αCD154 (*n* = 7), multi-DST-MHCII^Δ^+αCD154 (*n* = 6); for TEa cells, 1X-DST (*n* = 10), multi-DST+αCD154 (*n* = 7), multi-DST-MHCII^Δ^+αCD154 (*n* = 5). Percentages were normalized to those in T cells from mice immunized with 1×-DST set to 1 (dotted lines). 1×-DST% cytokine-positive mean ± SEM: TCR75 IFN-γ (49.69% ± 3.867%), ΤEa IFN-γ (28.99% ± 5.656%), TCR75 TNF (69.11% ± 2.519), TEa TNF (64.45% ± 5.838%). Statistics not depicted in the plots (1-way ANOVA): *P* < 0.001 between 1X-DST (dotted line) and multi-DST+αCD154 for both IFN-γ (**B**) and TNF (**C**) for both TCR75 and TEa cells; *P* < 0.001 between 1X-DST (dotted line) and multi-DST-MHCII^Δ^+αCD154 for both IFN-γ (**B**) and TNF (**C**) for TCR75 cells; *P* < 0.05 between (dotted line) and multi-DST-MHCII^Δ^+αCD154 for both IFN-γ (**B**) and TNF (**C**) for TEa cells. (**D**) CD44^hi^CD45.1^+^ tracer TCR-Tg cells were sorted from spleens and lymph nodes on day 35 and adoptively transferred into naive B6 secondary hosts. One day later, secondary hosts were immunized with B/c splenocytes and TCR-Tg cell expansion was measured in the spleen 5 days later, normalized to the average cell recovery from 1X-DST set to 1 for each experiment. For TCR75 cells: 1×-DST (*n* = 19), multi-DST+αCD154 (*n* = 11); for TEa cells: 1×-DST (*n* = 13), Multi-DST+αCD154 (*n* = 7). All data points represent a sample pooled from 1–2 mice, with lines indicating mean ± SEM. Data were analyzed by unpaired 2-tailed *t* test. **P <0.01, ****P < 0.0001.

Our combined results from TCR75, TEa, and OTII cells from tolerant mice suggested that persistently expressed cognate antigens drive more loss of function than more transiently expressed antigens. Thus, we investigated whether TEa cells could be made more hypofunctional upon CοΒ if exposure to their alloantigen was extended. To address this, naive untransplanted B6 mice seeded with tracer TCR75 or TEa cells were immunized with 1 injection of B/c splenocytes (1X-DST) or were treated with αCD154 (days 0, 7, and 14) along with repeated injections of B/c splenocytes every 48 hours for 35 days (multi-DST+αCD154) prior to functional analysis of the persisting tracer TCR-Tg cells (Figure 7A). Multi-DST+αCD154 successfully drove dysfunction not only of TCR75 but also of TEa cells, as determined by their impaired production of both IFN-γ and TNF when compared with memory T cells from DST-immunized mice (Figure 7, B and C). Of note, multi-DST+αCD154–dependent loss of cytokine production was detected whether cells were restimulated with anti-CD3/CD28 in vitro or with T cell–depleted F1 (B/cxB/6) splenocytes that express the cognate antigen (Supplemental Figure 4). Critically, loss of TEa function was dependent upon DST expression of MHCII, as injection of multi-DST+αCD154 using DST depleted of all MHCII^+^ cells (MHCII^Δ^-DST, starting on the second injection of multi-DST to enable a first injection of control DST to provide antigen for initial TEa activation) prevented acquisition of TEa but not TCR75 hypofunction (Figure 7, B and C). Depletion of MHCII-expressing cells was complete, as CFSE-labeled naive TEa cells transferred into hosts prior to 1X-MHCII^Δ^-DST failed to proliferate, in contrast with TEa cells exposed to 1X-control DST (Supplemental Figure 5, A–F). In addition to loss of cytokine-production capacity, control multi-DST+αCD154 led to a profound impairment in recall proliferation in both TCR75 and TEa cells when transferred and rechallenged in vivo in naive secondary hosts (Figure 7D). While the tolerizing effect of multi-DST+αCD154 remained significant 30 days after final DST injection in TCR75 and TEa cells for TNF and IFN-γ production, both TCR75 and TEa T cells had regained some TNF production at that time point, and because of the absence of a graft as a persistent source of K^d^, TCR75 cells had also recovered some IFN-γ production (Supplemental Figure 6). Together, these results indicate that increasing the duration of alloantigen exposure can lead to lasting, but not permanent, loss of function in TEa cells and show that TEa cells are not intrinsically resistant to developing hypofunction. These data also suggest a therapeutic avenue for increasing the robustness of donor-specific T cell hypofunction and potentially transplantation tolerance and hint at the fact that boosting tolerance may be necessary to ensure its persistence across a wide repertoire of allospecific T cells.

**Figure 8:**
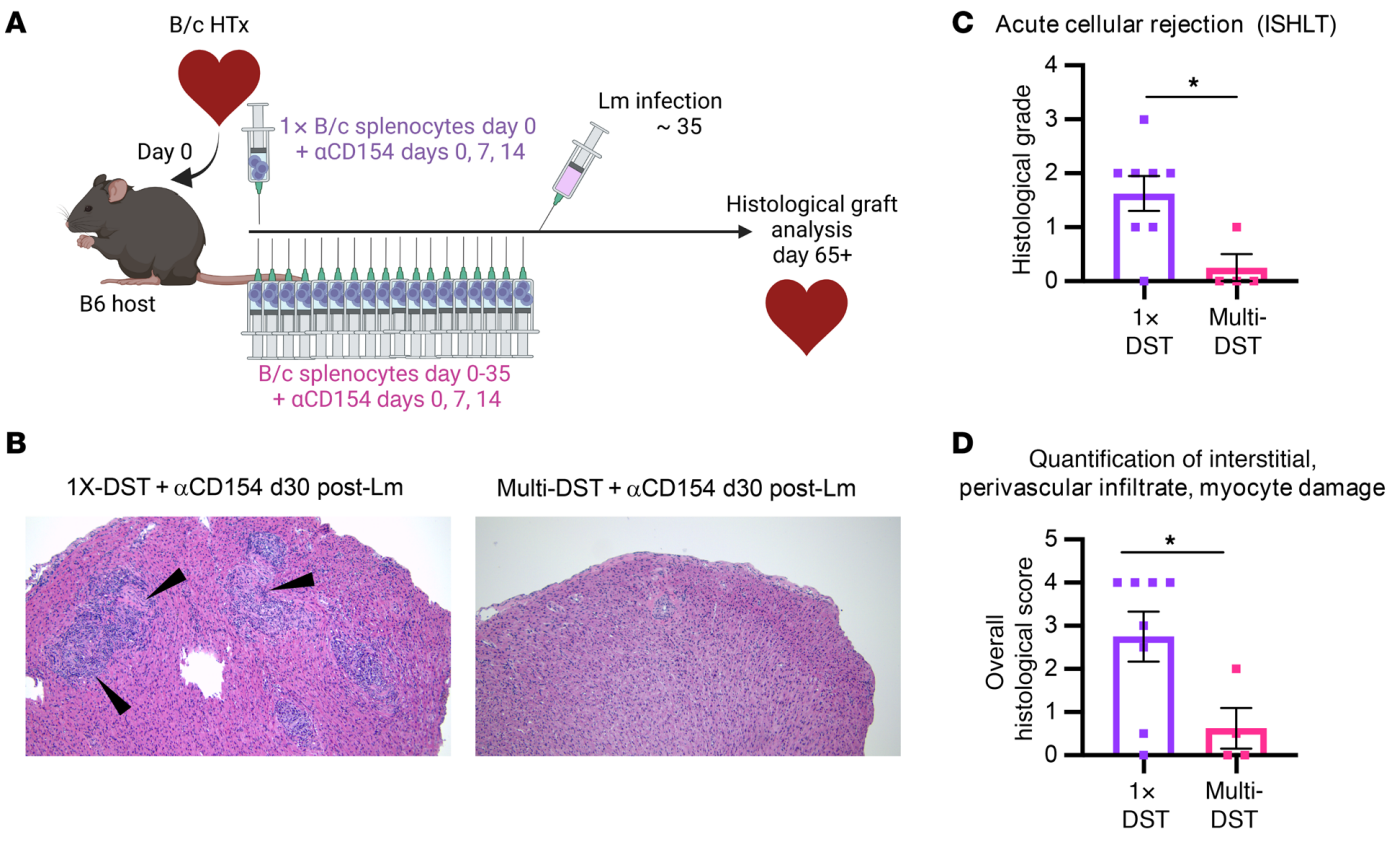
Prolonged exposure to alloantigen during αCD154 treatment protects heart grafts against post-Lm rejection. (**A**) Experimental design. B6 mice were transplanted with a B/c heart and tolerance was induced with αCD154 (days 0, 7, and 14) in all hosts and either a single injection of B/c splenocytes (1×-DST) or repeated injections of B/c splenocytes every 48 hours until day 35 (Multi-DST). Transplant hosts were then all infected with Lm on day 35, and grafts were analyzed a month after infection. (**B**) Representative histological images from mice described in **A**. Tissues were sectioned and stained with H&E. Original magnification, ×10 (with an infinity HD camera mounted on an Olympus microscope). (**C** and **D**) Myocardial tissue was examined and scored by an independent pathologist in a single-blinded manner using the International Society for Heart and Lung Transplantation (ISHLT) acute cellular rejection grading scale (48). 1X-DST+αCD154+Lm (*n* = 8); multi-DST+αCD154+Lm (*n* = 4). Data were compared using Mann-Whitney nonparametric 1-sided *t* test, with lines indicating mean ± SEM. *P <0.05.

Lm infection is capable of breaking or eroding stable heart allograft tolerance in infected tolerant hosts (5, 8). We hypothesized that multi-DST+αCD154 may increase the host’s protection from Lm-associated graft rejection, as a wider array of endogenous T cells would persistently encounter their cognate alloantigens, facilitating greater functional loss. To test this hypothesis, mice were transplanted with B/c heart allografts and tolerance was induced with 1X-DST+αCD154 or with multi-DST+αCD154 (Figure 8A). Mice were subsequently infected with Lm 35 days after transplantation, and heart grafts were evaluated 1 month after infection (i.e., 30 days after last injection of multi-DST). Mice exposed to multi-DST+αCD154 showed reduced rejection scores of their heart grafts after Lm infection when compared with mice exposed to 1X-DST+αCD154 as well as reduced immune cell infiltration and interstitial and perivascular inflammation and a noticeable reduction in tissue damage upon histological analysis (Figure 8, B–D). Thus, with prolonged exposure to a broader array of cognate alloantigens during αCD154 therapy, heart allografts are better protected from rejection following Lm infection.

## Discussion

In this study, we show that alloreactive Tconvs of different donor antigen specificities develop varied levels of dysfunction during CoB-induced transplantation tolerance and that the duration of expression of the different alloantigens following transplantation contributes to this heterogeneity. Allospecific T cells experiencing persistent antigen stimulation during tolerance induction, modeled by TCR75 cells, develop a phenotype more consistent with anergy/exhaustion and are more hypofunctional than T cells specific for transiently expressed alloantigens, such as TEa cells, or OTII cells transiently exposed to OVA. Cells that retain more function pose a greater threat to graft survival when tolerant hosts are exposed to inflammatory challenges, such as infections, which can upregulate alloantigen expression. However, these allospecific T cells can be made hypofunctional upon prolonged exposure to alloantigen in the form of multi-DST+αCD154. Most notably, this treatment was sufficient to protect tolerant grafts against Lm-mediated rejection.

It has been reported in various settings that T cells exposed to acute versus chronic antigens acquire different fates (26). Importantly, in chronic LCMV infection or cancer situations, exhausted T cells that are not terminally differentiated as determined by their retained expression of the stemness marker Tcf-1 can be reinvigorated (27). In contrast to infections, which are either acute (transient antigen) or chronic (persistent antigen), we show that an allograft *simultaneously* contains transient and persistent alloantigens resulting in heterogeneity of fates of donor-reactive T cells. Here, CD4^+^ T cells made hypofunctional by αCD154+DST were not reinvigorated by Lm infections despite their retained expression of Tcf-1 (Figure 3D), and instead, it was reactivation of nonhypofunctional CD4^+^ T cells by reexpression of transient antigen that led to rejection.

T cells recognizing intact donor MHCII directly on donor cells or crossdecorated on recipient APCs are anticipated to experience transient kinetics of stimulation similar to those of T cells recognizing donor MHCII peptides presented by recipient APCs because the cellular sources of intact donor MHCII are eliminated relatively shortly after transplantation (15). Furthermore, MHCII-derived alloantigens are likely not the only epitopes transiently expressed in donor cells during tolerance induction. Inflammation-induced polymorphic minor histocompatibility antigens, such as MHCI-related chain A (MICA) antigens, are similarly predicted to decline after resolution of ischemia/reperfusion injury. Additionally, T cells with low avidity for alloantigens, irrespective of their persistence, or T cells specific for low-density alloantigens may not be sufficiently stimulated to develop hypofunction during CoB treatment. Consequently, there is potentially a large population of allospecific T cells that do not experience the chronic stimulation needed to program hypofunction during tolerance induction. These cells may not reject the graft during stable tolerance due to insufficient expression of cognate antigen and/or control by Tregs but may escape suppression during inflammatory events. Indeed, in addition to inflammation potentially upregulating expression of certain alloantigens in the graft, IL-6, type I IFN, and TNF, cytokines produced during Lm infection have been shown to reduce susceptibility of Tconvs to Treg suppression (28) or attenuate Treg function (29, 30).

T cells specific for transiently expressed alloantigens that retain function pose a threat to the allograft when they are later reexposed to their cognate alloantigen, while T cells specific for alloantigens irreversibly downregulated shortly after transplantation are likely to be harmless. For T cells recognizing donor MHCII, we found that secondary alloantigen exposure can occur during an inflammatory challenge, such as Lm infection. We reason that these findings would translate to all vascularized grafts harboring donor-derived endothelial cells that survive long term after transplantation, as donor MHCII is upregulated on graft endothelial cells in response to IFN-γ, for example, during infection (31) or autoimmunity (32). We hypothesize expression of transiently expressed minor histocompatibility antigens such as MICA would also be upregulated during injury or inflammation.

The functional allospecific T cells we have identified may play an important role in the chronic rejection that occurs late after transplantation in patients on conventional immunosuppression (33–36). Prior work from our lab showed that TCR75 cells also developed hypofunction in transplanted mice treated with conventional immunosuppression that do not develop transplantation tolerance (12). In this setting, alloimmune responses still occurred late after transplantation, resulting in acute and chronic rejection possibly carried out by alloreactive T cells that retained functionality in these mice and in patients. Indeed, alloantibodies produced de novo after transplantation are often directed toward alloantigens predicted to exhibit downregulated expression following transplantation, but that can be induced later by inflammation. For example, de novo alloantibody production is more frequently directed toward donor MHCII than MHCI, and patients with class II DSA have worse outcomes (34, 35). Additionally, alloantibodies specific for the polymorphic stress-induced molecule MICA have been detected in transplant recipients and are associated with poor graft outcomes (36).

The concept of tolerizing the immune system with repeated exposure to antigen is reminiscent of clinical methods used to treat allergy patients. Patients who ingested low doses of oral allergens daily saw improved tolerance to their allergens (37, 38). This unresponsiveness was maintained for up to 4 years after the cessation of treatment (39, 40). Similarly, patients exposed weekly to grass-pollen allergens subcutaneously over the course of 3 years maintained prolonged clinical remission following the cessation of treatment (41, 42), implying that repeated exposure to antigen does not need to be maintained indefinitely for long-term disease-modifying effects and that tolerization is possible even after sensitization. These data could inform more clinical approaches for patient exposure to alloantigen, where patients could be given small doses of alloantigen to promote robust tolerance.

Our data show modest but significant (*P* < 0.05) recovery of cytokine production capability by multi-DST+αCD154–exposed TCR75 and TEa cells 1 month after last injection of multi-DST in untransplanted mice that lack the graft as an additional source of donor MHCI–derived peptide to maintain hypofunction of TCR75 cells. Tolerization may be longer lasting in the presence of a graft, as donor MHCI is constitutive and endothelial cells retain low levels of donor MHCII (Figure 5B), which may be sufficient to maintain hypofunction of multi-DST+αCD154–exposed donor MHCII–reactive T cells. Nevertheless, it is conceivable that the tolerogenic potential of multi-DST+αCD154 on T cells whose cognate antigen is transient in the graft will wane over time. One approach may be to reinduce/boost tolerance every so often with a regimen similar to multi-DST+αCD154. Alternatively, we have previously shown that the longer alloreactive T cells are exposed to their cognate antigen in the graft, the more profound the dysfunction becomes, with 60 days inducing greater loss of recall proliferation than 30 days (12). Thus, it may be that a longer multi-DST+αCD154 regimen may drive permanent hypofunction. These hypotheses will need to be tested in future experiments.

Our findings have additional implications for autoimmune diseases known for relapse and remittance. In Lewis rat models of experimental autoimmune uveitis, relapsing or monophasic disease onset is determined by the autoantigen used for induction (43). CD4^+^ T cells driving relapsing disease retain greater functionality when compared with CD4^+^ T cells in monophasic disease (44), perhaps because their cognate antigen is only transiently expressed. It may be possible to ameliorate relapsing disease by targeting these autoreactive T cells with repeated injections of antigen in combination with costimulation blockade. It is unclear how or whether persistent antigen exposure would treat or exacerbate models of remitting/relapsing autoimmunity that rely on epitope spreading, such as in autoimmune blistering diseases (45).

As efforts proceed within clinical transplantation toward inducing tolerance therapeutically and predicting which patients can safely undergo immunosuppression weaning without experiencing rejection, a key concern will remain whether transplant acceptance in these patients is durable. Reports in patients who have developed spontaneous operational tolerance or undergone concurrent renal and bone marrow transplantation indicate that we cannot yet guarantee permanent graft acceptance free from immunosuppression, even in patients who experience years of stable function after drug weaning. Understanding the mechanisms by which rejection can occur late after transplantation, both in tolerized patients and those treated with conventional immunosuppression, is therefore key to improving clinical outcomes for transplant recipients. We have identified a previously unappreciated source of persistent reactivity in the allospecific T cell repertoire that poses a substantial risk to graft survival. By reducing the effector function of these cells, we were able to prevent rejection of cardiac allografts after infection of tolerant hosts. Further work is needed to identify transiently expressed alloantigens in humans that can later become upregulated by inflammatory cues and to analyze the fate of T cells responding to those antigens late after transplantation. Our results suggest that prolonging exposure to alloantigen panels under the cover of costimulation blockade or matching donor and recipient alleles of key inflammation-induced alloantigens may increase the robustness of donor-specific tolerance and improve long-term graft survival in transplant recipients.

## Methods

### Mice.

B6 and B/c mice were purchased from Envigo RMS. TCR75 TCR-Tg mice obtained from R. Pat Bucy (Department of Pathology, University of Alabama at Birmingham, Birmingham, Alabama, USA) are specific for a peptide derived from K^d^ (donor MHCI) presented on I-A^b^ (host MHCII), and TEa TCR-Tg mice obtained from Alexander Rudensky (when at the Department of Immunology, University of Washington, Seattle, Washington, USA) are specific for a peptide derived from I-E^d^ (donor MHCII) presented on I-A^b^ (host MHCII). TCR75 and TEa mice were crossed with *Rag^–/–^* mice and CD45.1^+^ mice to generate TCR75/*Rag^–/–^*/CD45.1^+^ (TCR75) and TEa/*Rag^–/–^*/CD45.1^+^ (TEa) mice, respectively. OTII mice were obtained from the Jackson Laboratory and bred in-house. TGO mice (expressing a fusion protein comprising transferrin receptor transmembrane domain [T], GFP [G], and OVA_230-259_ [O] under the control of a tetracycline response element) were obtained from M. Rosenblum (Department of Dermatology, University of California at San Francisco, San Francisco, California, USA) and bred to R26-M2rtTA mice (encoding a mutant reverse tetracycline-controlled transactivator [rtTA] with low background activity in the absence of Dox) obtained from the Jackson Laboratory. Exposure of the resulting mice to Dox resulted in membrane-bound OVA upregulation in all tissues, while cessation of Dox downregulated OVA expression. Mice were age and sex matched when possible and housed under specific pathogen–free conditions.

### Heart transplantation and tolerance induction.

Cardiac transplantation was performed using a technique adapted from Corry et al. (46). For induction of tolerance, mice were treated with 500 to 600 μg of αCD154 (MR1, BioXCell) on days 0 (i.v.), 7, and 14 (i.p.) after transplantation and DST (i.v.) on day 0. B/c DST was prepared by homogenizing a single-cell suspension of splenocytes through a 40 μm filter. Each injection contained splenocytes from one-quarter to one-sixth spleen in 200 μL PBS. In all experiments where mice were treated with DST, day 0 injection was i.v. In mice treated with repeated DST injections every 48 hours, all injections after day 0 were i.p.

### Adoptive cell transfer.

In experiments where TCR-Tg tracer cells were seeded before transplantation or immunization, cells were isolated from the spleen and lymph nodes (inguinal, axillary, brachial, cervical, and mesenteric) of naive TCR75, TEa, or OTII mice and counted with an Accuri C6 or Fortessa flow cytometer (BD Biosciences); 5 × 10^4^ cells were injected i.v. in 200 μL of PBS up to 1 day before transplantation or before the first DST injection.

### Isolation of tracer TCR-Tg cells from adoptive transfer hosts.

Spleen and lymph nodes from primary hosts were harvested and homogenized 30 or more days following transplantation or first DST injection. Single-cell isolates were stained with anti-CD45.1-biotin (eBioscience) and incubated with streptavidin magnetic beads (Miltenyi Biotec) for magnetic enrichment with LS columns (Miltenyi Biotec) or AutoMACS (Miltenyi Biotec). In some experiments, cells from mice within the same experimental group were pooled after magnetic enrichment. Magnetically enriched cells isolated from positive selection were then stained with fluorophore-conjugated anti-CD45.1 (clone A20), anti-CD45.2 (clone 104), anti-CD4 (clone L3T4), anti-CD8 (clone Ly2), and anti-CD44 (clone IM7) (all from BioLegend) and sorted for CD45.1^+^CD4^+^CD44^hi^ cells on a FACSAria cell sorter (BD Biosciences). Cells were sorted into FBS, then washed and resuspended in PBS and subjected to further staining or functional analyses in vitro or in vivo.

### Bulk RNA-Seq acquisition and analysis.

Using the SMART-Seq v4 Ultra Low Input RNA-Seq kit from Takara Biosciences, we sorted 1,000 TCR75 cells directly into 1× lysis buffer with RNase inhibitor. We followed the manufacturer’s instructions, amplifying cDNA for 18 PCR cycles after first-strand synthesis. We then used the Nextera XT DNA Library Preparation Kit (Illumina) in combination with IDT DNA Unique Dual Indexes to create libraries for sequencing per the manufacturer’s instructions. The samples were sequenced using the Illumina NovaSeq platform with a 100 bp cassette and SP Flowcell. FAST-QC was performed on the resulting Fastq files to ensure the quality of the samples before moving on to alignment using the STAR splice-aware mapper and the GRCm39 mouse genome from Ensembl. A counts matrix was generated from the resultant BAM files using FeatureCounts. These raw counts were analyzed using the DESeq2 package on R to identify differentially expressed genes and normalized gene counts.

### Cell preparation and staining for flow cytometry.

Flow cytometry was performed in the CAT Facility (RRID: SCR_017760) at the University of Chicago. Sorted TCR-Tg cells or unenriched spleen and lymph node cells were homogenized, filtered, and stained with a fixable LIVE/DEAD dye (Invitrogen). Cells were then stained with fluorophore-conjugated antibodies from BioLegend to CD4 (L3T4), CD8 (Ly2), CD44 (IM7), CD127 (A7R34), CD73 (TY/11.8), and B220 (RA3-6B2) or Invitrogen to PD-1 (J43). Surface-stained cells were then fixed with the FoxP3 fixation permeabilization buffer kit (eBioscience) for 15 to 30 minutes at room temperature (RT) and washed with 1× permeabilization buffer. Some samples were intracellularly stained with antibodies from Invitrogen to Ki67 (SolA15) or anti-FoxP3 (FJK-16s) for 30 minutes at RT, washed with permeabilization buffer, and analyzed by flow cytometry. For experiments involving spectral flow cytometry, all samples were analyzed using a Cytek Aurora Flow Cytometer (5 lasers, 16UV-16V-14B-10YG-8R). The fluorophore-conjugated antibodies/dyes used for spectral flow analysis included, from Invitrogen, Fixable LIVE/DEAD stain (LIVE/DEAD Fixable Blue Dead Cell Stain Kit), CD44 (12A5), FoxP3 (FJK-16s), and Tox (TXRX10); from BioLegend, Tcf-1 (7F11A10), CD45.1(A20), PD-1 (29F.1A12), Slamf6 (330-AJ), CD62L (MEL-14), CD74 (TY/11.8), Lag3 (C9B7W), and Satb1 (O96C6); from BD, Tigit (IG9), CD90.2 (53-2.1), CD4 (GK1.5), CD8 (5H10-1), TER-119 (TER-119), CD19 (1D3), CD11c (N418), F4/80 (T45-2342), NK1.1 (PK136), CTLA4 (UC10-4F10-11), and FR4 (12A5); and from Santa Cruz Biotechnology Inc., NFATc1 (7A6).

To identify DCs, 5 × 10^6^ unenriched splenocytes were stained with 1:1,000 fixable LIVE Aqua LIVE/DEAD dye (Invitrogen) for 20 to 30 minutes at RT in the dark, surface antibodies (BioLegend) to CD45.2 (clone 104), CD19 (clone 6D5), CD11c (clone N418), and I-A/I-E (clone M5/144.15.2), and to YAe (eBioY-Ae) (Invitrogen) for 10 minutes at RT in the dark, washed, resuspended in FACs buffer and analyzed with the LSR Fortessa. DCs/APCs were isolated by gating on live, CD45.2^+^CD19^–^CD11c^+^I-A/I-E^+^ events.

Cardiac grafts were isolated from recipient mice and washed with heparin/1× HBSS (Thermo Fisher). Cardiac tissue was then cut to small pieces and digested with 400 U/mL of collagenase IV (MilliporeSigma), 0.01% DNase I (MP Biomedicals), and 10 mM HEPES (Thermo Fisher) in HBSS for 40 minutes while incubated at 37°C. The tissue solution was homogenized by passing through a 40 μm filter cup, washed, and quenched with an excess of complete DMEM (5% FBS, 1% HEPES, 1% nonessential amino acids, 1% penicillin-streptomycin, 1% l-glutamine, 0.0004% 14M β-Mercaptoethanol). Cells were washed with 1× PBS, then stained with 1:1,000 fixable LIVE Aqua LIVE/DEAD stain (Invitrogen) for 20 to 30 minutes at RT in the dark, followed by surface antibodies by BioLegend to CD45.2 (clone 104) and I-A/I-E (clone M5/144.15.2) and to H2:K^d^ (clone SF1-1.1.1, Invitrogen), and CD31 (clone MEC13.3, BD) for 10 minutes at RT in the dark, washed, and resuspended in FACS buffer and analyzed with the LSR Fortessa. Donor-derived endothelial cells were isolated by gating on live CD45.2^–^CD31^+^H2:K^d+^ events.

For experiments involving MHCII depletion (MHCII^Δ^), whole spleens from B/c mice were resuspended (10^6^ cells/mL). The spleens were depleted of MHCII-expressing cells using the following biotinylated antibodies (all from BioLegend): B220 (RA3-6B3), CD11b (M1/70), F4/80 (BMB), CD11c (N418), and MHCII (M5/114.15.2). Following a 10-minute incubation period, magnetized streptavidin beads (Miltenyi Biotec) were added (37.5 μL/mL sample), samples were incubated for an additional 10 minutes, and then MHCII-positive cells were removed using a magnet. Depletion was verified by flow cytometry. For the group receiving MHCII^Δ^ multi-DST+αCD154, control DST (MHCII-sufficient) was administered at the first injection to allow initial cognate antigen recognition in the absence of a graft, and subsequent injections were MHCII^Δ^.

### CFSE dilution assay of proliferation.

TCR-Tg cells were stained with a fixable CFSE dye (Invitrogen) as previously described (47). Briefly, no more than 5 × 10^6^ T cells were resuspended in 1 mL PBS and 5% FBS and vortexed, and a 1 mL solution of 8 to 10 μM CFSE was added drop by drop to the cell solution for a final concentration of 4 to 5 μM CFSE. Cells were incubated for 5 minutes at RT prior to being quenched in 5 mL 5% FBS medium. Unstimulated CFSE-labeled cells were cultured at 37°C with 1 ng/mL human IL-7 (PeproTech) as flow cytometry single stains. Cells were adoptively transferred to mice on day 0 and recovered on day 4 after injection.

### In vitro stimulation for cytokine production.

U-bottom tissue culture plates were coated for 90 minutes at 37°C or overnight at 4°C with 5 μg/mL anti-CD3 (2C11) and 1 μg/mL anti-CD28 (PV.1) (Fitch Monoclonal Facility). Tracer TCR-Tg cells sorted from primary hosts were plated per well and incubated for 16 to 24 hours at 37°C in 5% CO_2_. For each individual experiment, all wells were plated with the same number of cells. In some experiments, 1 × 10^5^ splenocytes from a naive B6 mouse were cocultured as filler cells with TCR-Tg cells to promote viability. Unstimulated controls were plated in uncoated wells with 1 ng/mL human IL-7 (PeproTech). For APC coculture stimulation, splenocytes from an F1 (B6 × B/c) mouse were depleted of T cells using anti-CD4 (GK1.5) and anti-CD8 (2.43) biotinylated antibodies (Invitrogen) and anti-biotin magnetic beads (Miltenyi Biotec). T-depleted splenocytes were then cultured at a concentration of 2 × 10^6^ cells/mL in complete DMEM with 2 μg/mL LPS in a 6-well tissue culture plate at 37°C overnight. Stimulated APCs (100 μL) were added to each well with TCR-Tg cells (100 μL) for incubation as described above. Two hours after plating, brefeldin A (BioLegend) was added to all wells. After stimulation, cells were stained with fixable viability dye (Invitrogen) and then surface stained with fluorophore-conjugated anti-CD4, anti-CD8, anti-CD45.1, anti-CD45.2, and anti-CD44. Cells were then fixed and permeabilized with the FoxP3 Fixation Permeabilization Buffer Kit (eBioscience) and stained with fluorophore-conjugated BioLegend anti–IFN-γ (XMG1.2) and anti-TNF (MP6-XT22) for 30 minutes at RT or overnight at 4°C before washing with permeabilization buffer and being analyzed by flow cytometry.

### Evaluation of recall expansion.

TCR-Tg cells (5 × 10^2^–6 × 10^3^) were injected i.v. into secondary naive B6 hosts immunized 24 hours later with B/c DST i.v. Within each experiment, the number of transferred cells was similar between mice. Cell concentration was confirmed by counting on either an Accuri C6 flow cytometer or an LSR 4 to 12 flow cytometer using CountBright Plus Absolute Counting Beads (Invitrogen) prior to injection. Five days after DST, 5 × 10^6^ splenocytes were isolated from the secondary hosts, then stained with a viability dye and fluorophore-conjugated anti-CD4, anti-CD8, anti-CD45.1, and anti-CD45.2. The number of CD45.1^+^CD4^+^ cells was calculated per mouse.

### Statistics.

See Supplemental Methods.

### Study approval.

All animal experiments were approved by the University of Chicago’s Institutional Animal Care and Use Committee. The University of Chicago’s animal care and use program is accredited by the Association for Assessment and Accreditation of Laboratory Animal Care International, and animals were maintained in accordance with the *Guide for the Care and Use of Laboratory Animals* (National Academies Press, 2011).

### Data availability.

RNA-Seq data are available in the ArrayExpress database (http://www.ebi.ac.uk/arrayexpress) following MINSEQE guidelines (E-MTAB-13333). R code used to analyze the data and create plots can be accessed on GitHub at https://github.com/amcassano/PersistenceOfAntigen.git Values for all data points in graphs are reported in the Supporting Data Values file.

## Author contributions

CMM and JBA performed experiments and wrote the manuscript. PW, SZX, and MLM performed experiments. YW, LC, and DY performed transplants. AC, RMC, GH, and DA assisted with experiments. HS analyzed histology. ZJZ and ASC provided intellectual and technical support and edited the manuscript. MLA conceived and oversaw the project, helped interpret data, and cowrote and edited the manuscript. ASC contributed to the data interpretation and edited the manuscript. Co-first authorship was determined by CMM initiating the project and writing the first draft of the manuscript.

## Supplementary Material

Supplemental data

Supporting data values

## Version 1 09/07/2023

In-Press Preview

## Version 2 11/01/2023

Electronic publication
