# Supplementary material for: Heterogeneity in allospecific T cell function in transplant-tolerant hosts determines susceptibility to rejection following infection
Source: J Clin Invest. 2023 Nov 1;133(21):e168465. doi: 10.1172/JCI168465 (PMC10617766; doi:10.1172/JCI168465)
Supplement: Supplemental data [file jci-133-168465-s143.pdf]

Supplemental Materials

Supplemental Methods

In vivo Lm infection:

An overnight culture of Lm engineered to express GFP (Lm) (18) was diluted 1:50 in brain heart infusion broth (BD Biosciences) supplemented with 10µl/ml chloramphenicol (Sigma) and grown until OD_{600} was within the log-phase of the Lm growth curve (~2hr). OD_{600} was used to calculate colony-forming units (CFU)/ml. Mice were infected i.p. with Lm ($8x10^5$-$1x10^6$ CFUs in 400µl per mouse) as this dose resulted in the highest rejection rate with minimal lethality. The pre- and post-infection inoculum was plated to confirm dosage. Graft survival was monitored twice per week after infection. A subset of mice received ampicillin (25mg/100µl in 1xPBS i.p.) 48-72hr post-infection to prevent Lm morbidity.

Histology:

Heart allografts were removed, weighed, halved laterally, and fixed in 10% neutral buffered formalin at RT for 36-48h. Tissues were sectioned, then stained with haematoxylin and eosin. Slides were imaged at 10x or 20x magnification with an infinity HD camera mounted on an Olympus microscope (model# BX45TF). Myocardial tissue was examined and scored by an independent pathologist in a single-blinded manner using the International Society for Heart and Lung Transplantation Acute Cellular Rejection (ISHLT ACR) grading scale, only analyzing the myocardium tissue and ignoring tissue near where the graft was stitched and anastomosed (20). Myocardial tissue was also examined for the extent of interstitial infiltrate (mild, moderate, severe), the absence/presence of perivascular infiltrate, and myocyte damage/necrosis (focal, diffuse, or absent), and quantified on a 4-point scale (0-1pt interstitial infiltrate, 0-1pt perivascular infiltrate, 0-2pt myocyte damage/necrosis).

Data and statistical analysis:

Flow cytometry data were analyzed using FlowJo (Version 10.9.0, Tree Star, Ashland, Oregon). Spectral flow cytometry analysis was performed using the FlowJo plugins DownSample (Version 3.3.1), UMAP

(Version 3.3.4), and FlowSOM (Version 3.0.18) and using R and the R package fmsb. Samples were first gated on live single cells, then on TCR-Tg conventional T cells ($CD90.2^{+}CD45.1^{+}CD4^{+}Foxp3^{-}$). The TCR-Tg Tconv populations from each condition were concatenated into a single FCS file, then 2000 cells per condition were randomly selected using DownSample to ensure equal representation of each condition in the final UMAP analysis. Subpopulations from all conditions were concatenated together, with the addition of a keyword to enable condition identification. UMAP was performed on this concatenated population with nearest neighbors set to 15 and the minimum distance set to 0.5. FlowSOM analysis was performed with number of clusters set to 6. The MFI of each marker for the TCR-Tg Tconv populations was exported for each sample and used in conjunction with R package fmsb to construct radar plots. Flow cytometry samples were gated on live single cells before analysis. Statistical analyses were performed where appropriate using GraphPad Prism (GraphPad, La Jolla, California). Each statistical test is listed in the figure legends.

Quantitative variables with a gaussian distribution are presented with mean +/- standard error of the mean or +/- standard deviation as indicated. Quantitative variables with a non-gaussian distribution are presented with median (P25-P75).

A p-value of <0.05 was considered statistically significant. *p<0.05, **p<0.01, ***p<0.001, ****p<0.0001. ns, not significant.

Supplemental Figures

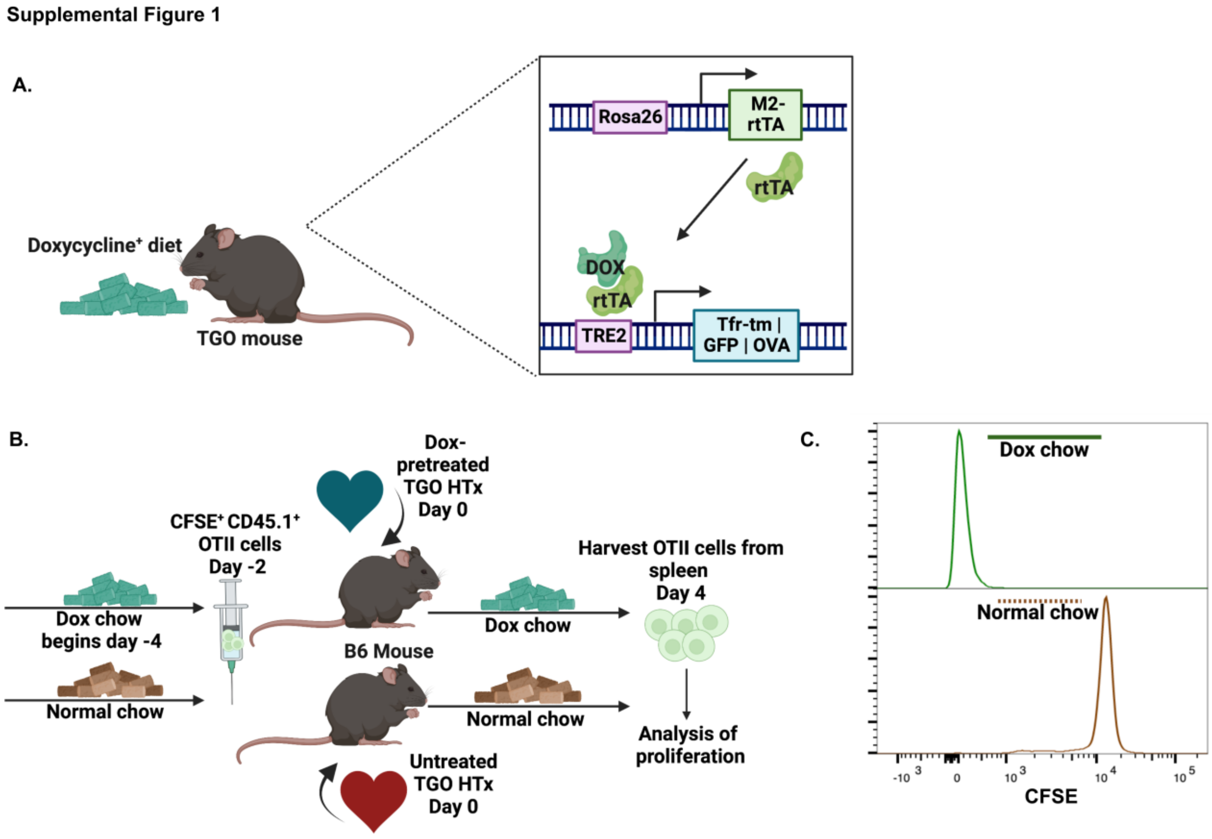

Supplemental Figure 1- Dox chow results in OVA expression by TGO^{+} hearts. A. Mouse model. Mice expressing a transferrin transmembrane domain-GFP-OVA fusion protein under the control of a tetracycline-response element (TRE2) were crossed to mice expressing the reverse tetracycline transactivator M2-rtTA under control of the Rosa promoter to enable rtTA expression in all cells. **B.** Experimental model. B6 recipients were adoptively transferred with CFSE-labeled CD45.1^{+}CD4^{+}*Rag*$^{-/-}$ OTII T cells (OTII) and given TGO heart transplants from donor on doxycycline (Dox)-containing diet or control chow. Recipients of OVA^{+}TGO hearts received Dox chow whereas recipients of OVA^{-}TGO hearts were kept on normal chow. OTII cell proliferation was evaluated in the spleen 4 days after transplantation.**C.** Representative plots of CFSE dilution of OTII cells confirm OVA expression and presentation in hosts of TGO heart allografts when donors and recipients are kept on Dox chow.

Supplemental Figure 2

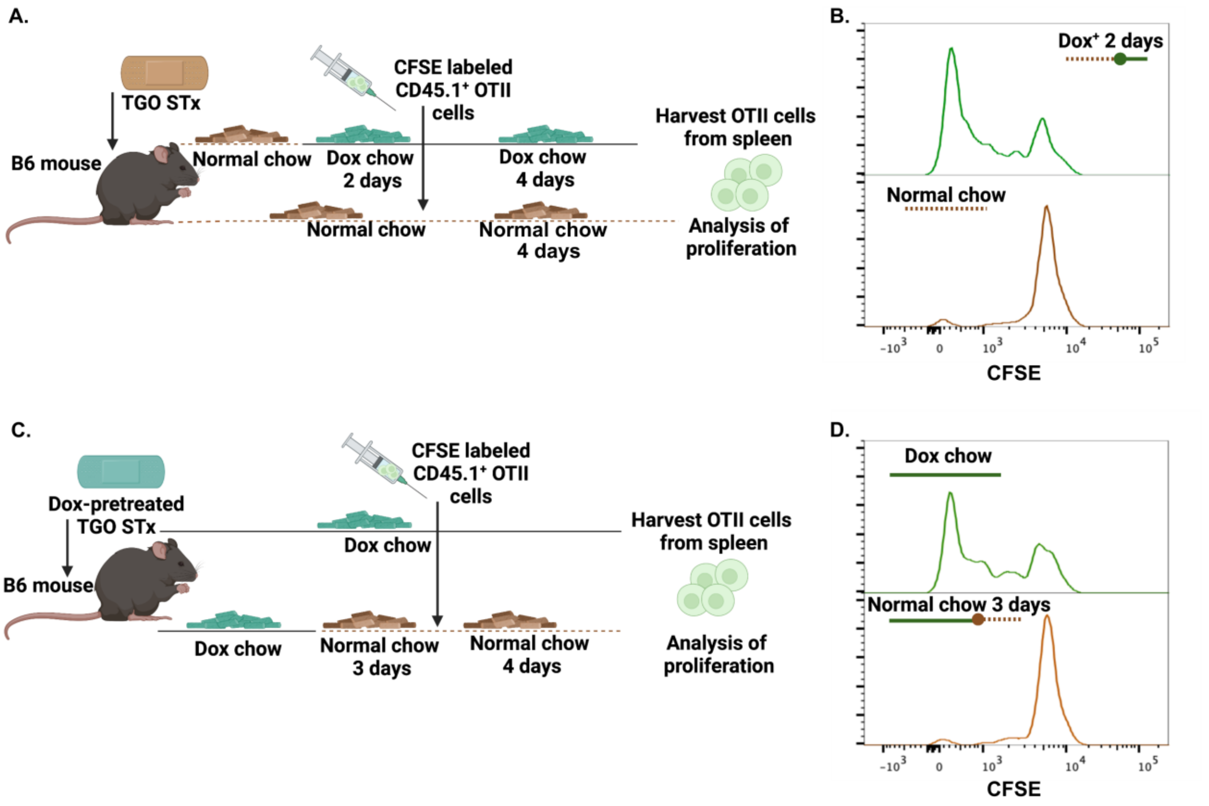

Supplemental Figure 2- Dox chow cessation extinguishes OVA expression in TGO^{+} grafts.
A. Experimental model. B6 recipients were given a TGO skin transplants. Some mice were exposed to a Dox-containing diet two days prior to adoptive transfer of CFSE-labeled OTII cells. Control mice were never exposed to a Dox-containing diet. OTII cells were reisolated from skin-draining lymph nodes 4 days post adoptive transfer.
B. Representative plots confirming that a Dox-containing diet results in OTII proliferation, evaluated by CFSE dilution in CD45.1^{+}CD4^{+}OTII cells.
C. Experimental model. B6 recipients were given a TGO skin transplant pre-exposed in donors to a Dox-containing diet. Some mice were returned to a normal chow diet three days prior to adoptive transfer of CFSE-labeled OTII cells. Control mice were continuously exposed to a Dox-containing diet. OTII cells were reisolated from skin-draining lymph nodes 4 days post adoptive transfer.
D. Representative plots confirming that stopping a Dox-containing diet prevents OTII proliferation, evaluated by CFSE dilution in CD45.1^{+}CD4^{+}OTII cells.

Supplemental Figure 3

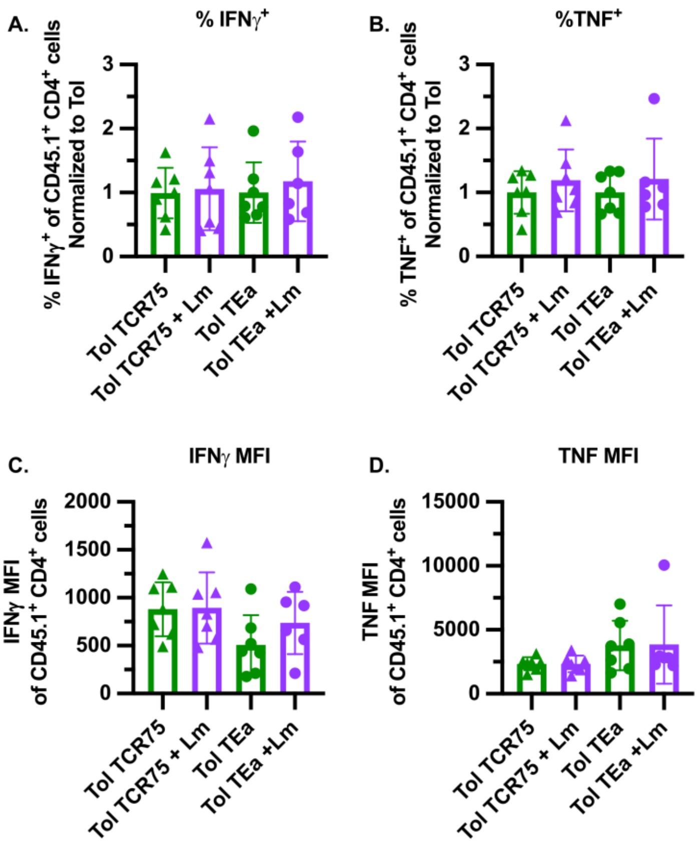

Supplemental Figure 3- Lm infection in tolerant hosts does not result in increased cytokine production by either TCR75 or TEa cells.

TCR75 or TEa cells were adoptively transferred into congenic B6 recipients prior to transplantation with a B/c heart allograft and treatment with CoB to induce tolerance in all hosts. After 35+ days, a subset of mice was infected with Lm. 4-8 days post-infection, CD45.1^{+} T cells were recovered, counted, and analyzed by flow cytometry.

A, B. Percent IFNγ (A) and TNF (B) producing cells out of CD45.1^{+}CD4^{+} cells recovered and upon restimulation with anti-CD3/CD28 *in vitro* in the presence of brefeldin A. TCR75 results normalized to Tol TCR75, and TEa results normalized to Tol TEa.

C, D. Amount of IFNγ (C) and TNF (D) produced on a per-cell basis measured by mean fluorescence intensity (MFI).

Tol TCR75 (n=7), Tol TCR75+Lm (n=7), Tol TEa (n= 7), Tol TEa +Lm (n=6).

Data were compared by unpaired 2-tailed t-test either comparing “Tol TCR75” versus “Tol TCR75 + Lm” or “Tol TEa” versus “Tol TEa + Lm groups”. $p<0.05$ is considered significant.

Supplemental Figure 4

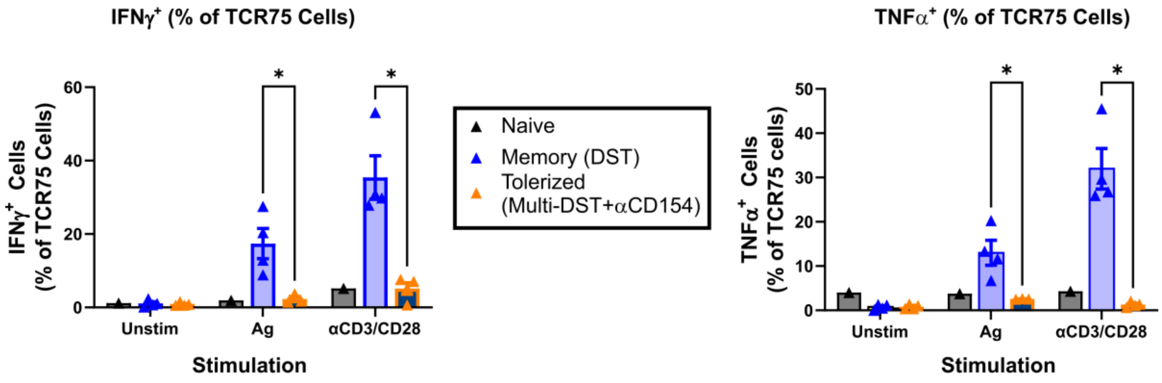

Supplemental Figure 4- Similar reduction of cytokine production following Muti-DST+αCD154 in TCR75 cells upon antigen or anti-CD3/CD28 restimulation. TCR75 cells were adoptively transferred into B6 recipients prior to exposure to one dose of B/c splenocytes or the Muti-DST+αCD154 regimen described in Figure 7. Following Muti-DST+αCD154, TCR75 cells were recovered by sorting for $CD45.1^+CD4^+$ cells. Sorted TCR75 cells were cultured overnight in the presence of brefeldin A either in co-culture with LPS-stimulated, T-depleted F1 (B6 x B/c) splenocytes (which can present TCR75 cognate antigen) or with plate-bound anti-CD3/CD28. Percent of IFNγ (left) and TNF (right) produced by TCR75 cells as assessed by flow cytometry is shown. The average of 1-3 technical replicates is plotted per symbol. Data were compared by unpaired Welch's t-test. Memory TCR75 (n=4), Tol TCR75 (n=4).

Supplemental Figure 5

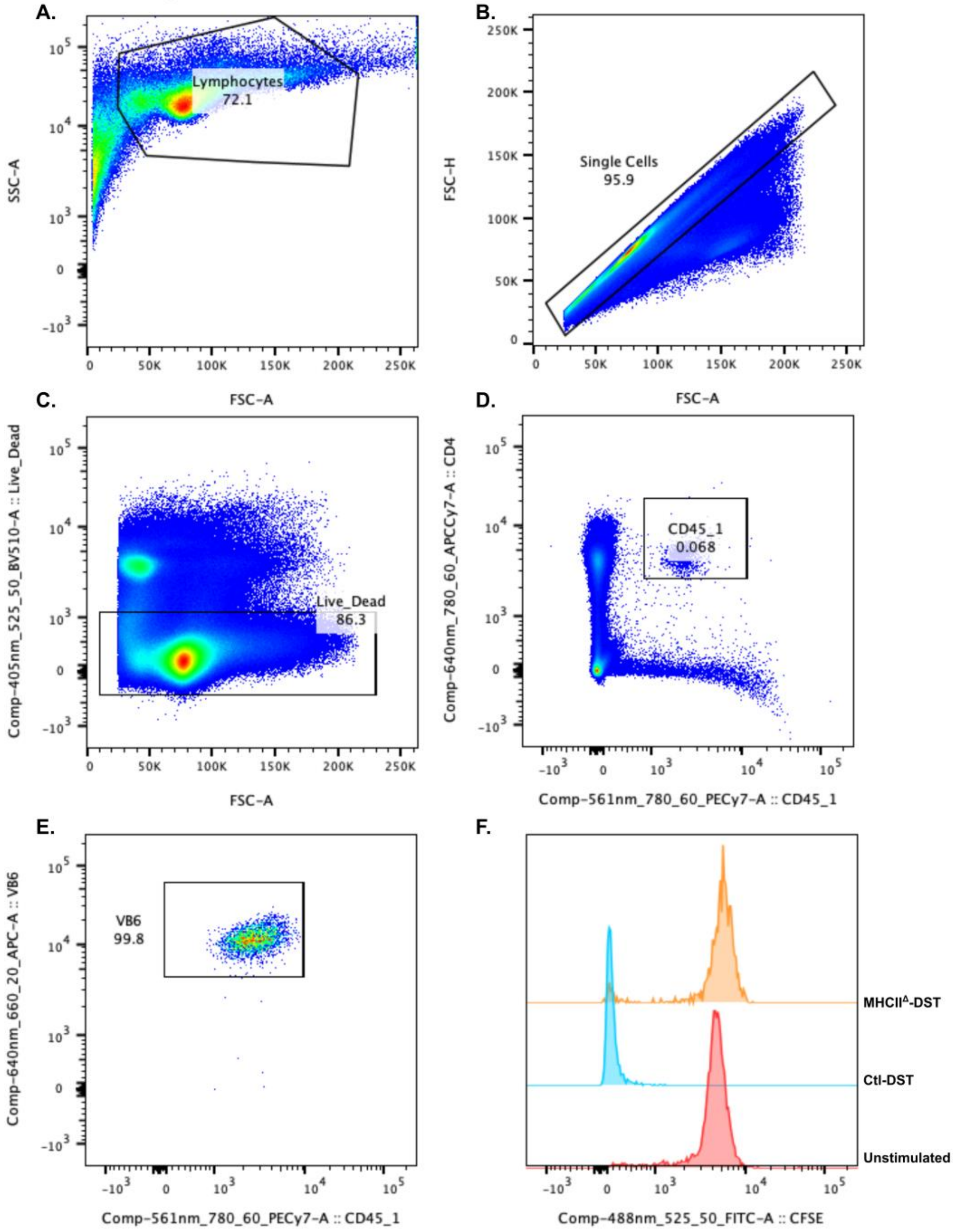

Supplemental Figure 5- MHCII-depletion protocol of DST prevents naïve TEa T cell proliferation. CFSE-labeled TEa cells were adoptively transferred into B6 hosts prior to injection of B/c splenocytes. Splenocytes were either unmanipulated (Ctl-DST) or were depleted for MHCII (MHCII$^{\Delta}$-DST). Three days post-injection of splenocytes, TEa cells were recovered and their CFSE dilution was assessed. **A-E** Gating strategy for TEa T cells *ex vivo*. **F.** TEa proliferation (CFSE dilution) after *in vivo* exposure to Ctl-DST versus MHCII$^{\Delta}$-DST, to verify elimination of donor MHC Class II-derived antigen, or in the absence of DST as unstimulated controls.

Supplemental Figure 6

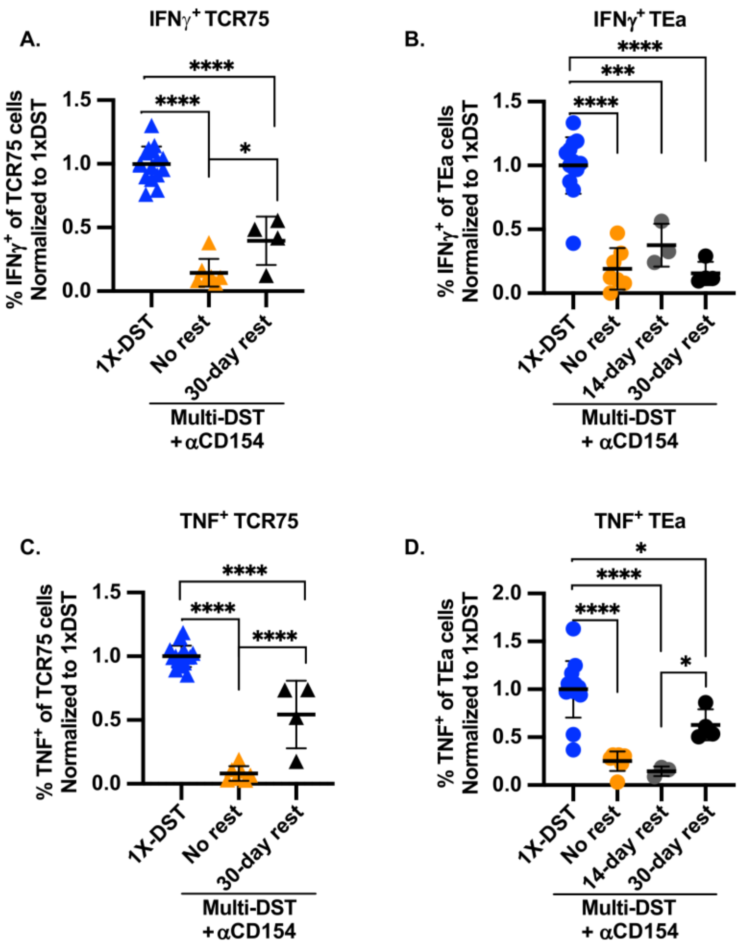

Supplemental Figure 6- Multi-DST+αCD154 exposure does not permanently prevent T cell function. Following Multi-DST+αCD154 exposure as described in Figure 7A, B6 mice were given periods of rest for either 14 or 30 days. (**A-D**) Percentage of TCR-Tg cells producing IFNγ (A, B) and TNF (C, D) following cell isolation from spleen and lymph nodes of primary hosts on day 14 or 30 of rest post last Multi-DST injection and restimulation overnight with anti-CD3/CD28 in the presence of brefeldin A. Experimental samples for 1XDST and Multi-DST+αCD154 are the same as depicted in Figure 7. Error bars represent Mean+/-SD. Data were compared by one-way ANOVA with Bonferroni correction for multiple pairwise comparisons.
